# Single-cell transcriptomic dissection of the cellular and molecular events underlying the triclosan-induced liver fibrosis in mice

**DOI:** 10.1186/s40779-023-00441-3

**Published:** 2023-02-22

**Authors:** Yun-Meng Bai, Fan Yang, Piao Luo, Lu-Lin Xie, Jun-Hui Chen, Yu-Dong Guan, Hong-Chao Zhou, Teng-Fei Xu, Hui-Wen Hao, Bing Chen, Jia-Hui Zhao, Cai-Ling Liang, Ling-Yun Dai, Qing-Shan Geng, Ji-Gang Wang

**Affiliations:** 1 Department of Nephrology, Shenzhen Key Laboratory of Kidney Diseases, and Shenzhen Clinical Research Centre for Geriatrics, Shenzhen People’s Hospital, the First Affiliated Hospital, Southern University of Science and Technology, Shenzhen, 518020 China; 2 Department of Urology, Shenzhen People’s Hospital, the First Affiliated Hospital, Southern University Science and Technology, the Second Clinical Medical College, Jinan University, Shenzhen, 518020 China; 3 Integrated Chinese and Western Medicine Postdoctoral Research Station, Jinan University, Guangzhou, 510632 China; 4 Artemisinin Research Center, and Institute of Chinese Materia Medica, China Academy of Chinese Medical Sciences, Beijing, 100700 China; 5 Guangdong Provincial Key Laboratory of New Drug Screening, School of Pharmaceutical Sciences, Southern Medical University, Guangzhou, 510515 China; 6 Center for Reproductive Medicine, Dongguan Maternal and Child Health Care Hospital, Southern Medical University, Dongguan, 523125 Guangdong China

**Keywords:** Triclosan, scRNA-seq, Liver fibrogenesis, Hepatic stellate cell

## Abstract

**Background:**

Triclosan [5-chloro-2-(2,4-dichlorophenoxy) phenol, TCS], a common antimicrobial additive in many personal care and health care products, is frequently detected in human blood and urine. Therefore, it has been considered an emerging and potentially toxic pollutant in recent years. Long-term exposure to TCS has been suggested to exert endocrine disruption effects, and promote liver fibrogenesis and tumorigenesis. This study was aimed at clarifying the underlying cellular and molecular mechanisms of hepatotoxicity effect of TCS at the initiation stage.

**Methods:**

C57BL/6 mice were exposed to different dosages of TCS for 2 weeks and the organ toxicity was evaluated by various measurements including complete blood count, histological analysis and TCS quantification. Single cell RNA sequencing (scRNA-seq) was then carried out on TCS- or mock-treated mouse livers to delineate the TCS-induced hepatotoxicity. The acquired single-cell transcriptomic data were analyzed from different aspects including differential gene expression, transcription factor (TF) regulatory network, pseudotime trajectory, and cellular communication, to systematically dissect the molecular and cellular events after TCS exposure. To verify the TCS-induced liver fibrosis, the expression levels of key fibrogenic proteins were examined by Western blotting, immunofluorescence, Masson’s trichrome and Sirius red staining. In addition, normal hepatocyte cell MIHA and hepatic stellate cell LX-2 were used as in vitro cell models to experimentally validate the effects of TCS by immunological, proteomic and metabolomic technologies.

**Results:**

We established a relatively short term TCS exposure murine model and found the TCS mainly accumulated in the liver. The scRNA-seq performed on the livers of the TCS-treated and control group profiled the gene expressions of > 76,000 cells belonging to 13 major cell types. Among these types, hepatocytes and hepatic stellate cells (HSCs) were significantly increased in TCS-treated group. We found that TCS promoted fibrosis-associated proliferation of hepatocytes, in which Gata2 and Mef2c are the key driving TFs. Our data also suggested that TCS induced the proliferation and activation of HSCs, which was experimentally verified in both liver tissue and cell model. In addition, other changes including the dysfunction and capillarization of endothelial cells, an increase of fibrotic characteristics in B plasma cells, and M2 phenotype-skewing of macrophage cells, were also deduced from the scRNA-seq analysis, and these changes are likely to contribute to the progression of liver fibrosis. Lastly, the key differential ligand-receptor pairs involved in cellular communications were identified and we confirmed the role of GAS6_AXL interaction-mediated cellular communication in promoting liver fibrosis.

**Conclusions:**

TCS modulates the cellular activities and fates of several specific cell types (including hepatocytes, HSCs, endothelial cells, B cells, Kupffer cells and liver capsular macrophages) in the liver, and regulates the ligand-receptor interactions between these cells, thereby promoting the proliferation and activation of HSCs, leading to liver fibrosis. Overall, we provide the first comprehensive single-cell atlas of mouse livers in response to TCS and delineate the key cellular and molecular processes involved in TCS-induced hepatotoxicity and fibrosis.

**Supplementary Information:**

The online version contains supplementary material available at 10.1186/s40779-023-00441-3.

## Background

Triclosan (TCS) is an antimicrobial agent that has been widely used worldwide for approximately 50 years since its first introduction in hospital settings [1, 2]. Although TCS has been banned from use in consumer antiseptic soap and hand sanitizer products by the US Food and Drug Administration (FDA) [3], it remains present in many readily accessible consumer products for daily use, such as toothpaste, mouthwash, shampoo, deodorant and cosmetics [4]. TCS is commonly added at a concentration of 3.5–17.0 mmol/L (i.e., 0.1–0.5%) [5]. Depending on the exposure route, TCS is absorbed into the human body through the skin or oral mucosa, and is detectable in human urine, nails, blood and breast milk [6, 7]. A recent study in China has reported detectable TCS in the urine and nails of 69–80% of participants [8]. The levels of TCS in the human body differ depending on the exposure sites, concentration/amount of exposure and type of exposure. Exposure to TCS-containing toothpaste for 14 d has been found to increase the TCS plasma concentration from 0.009–0.81 to 26–296 ng/g [9]. Use of 15 ml mouthwash containing as little as 0.03% TCS twice daily has been found to result in a plasma concentration of TCS plateauing at 74.5–94.2 ng/ml within as little as 2 d [10]. In humans, the liver is the main organ of TCS accumulation and metabolism [11]. TCS present at low levels is quickly detoxified by glucuronidation and sulfonation through catalysis by glucuronosyltransferases and sulfotransferases [12]. The potential risks of TCS on human health have drawn the public’s attention, and have been a subject of active debate and study in recent years [4, 13–15].

Long-term exposure to TCS may potentially cause various disorders, including gut microbiome impairment [16], induction of colonic inflammation and colitis-associated colon tumorigenesis [17], neurobehavioral toxicity [18, 19] and increased incidence of bone diseases [20]. The hepatotoxicity of long-term exposure of TCS has also been reported, thus promoting not only nonalcoholic steatohepatitis (NASH) but also liver tumorigenesis [21, 22]. TCS-induced hepatotoxicity has been attributed to the activation of constitutive androstane receptor and peroxisome proliferator activated receptor α (PPARα), and the promotion of liver fibrogenesis [23, 24]. Months-long TCS exposure has been found to impair lipid homeostasis and accelerate liver damage in mice [25]. However, the underlying cellular and molecular mechanisms of hepatotoxicity induced by TCS exposure, particularly at the initiation stage, remain largely unknown.

In contrast to traditional bulk RNA sequencing, single cell RNA sequencing (scRNA-seq) can unveil the transcriptional heterogeneity of cells and tissues with high accuracy and reproducibility [26]. This technique has been used in deciphering the toxicity profiling of many hazardous substances in humans and other animals [27, 28]. In addition, scRNA-seq has been used in studies of liver diseases, including liver cirrhosis [29], NASH [30] and hepatocellular carcinoma [31]. The cellular landscape of the liver has also been recently described by scRNA-seq [32].

In this study, a relatively short-term TCS exposure mouse model was established. To explore the potential hepatotoxicity mechanism of TCS at the single cell level, we used scRNA-seq to comprehensively profile the changes in gene expression and the modulation of different cell types in the liver of these mice.

## Methods

### Animals and drug treatment

All animal experimental procedures were approved by the Animal Care and Use Committee at Shenzhen People’s Hospital (No. AUP-210901-DLY-0001-01). Eighteen male C57BL/6 mice (5 weeks old) were purchased from GemPharmatech (Guangdong, China) and maintained for 7 d for adaption. The mice were randomly divided into three groups (control, TCS-100 and TCS-200, with 6 mice per group), which were treated with corn oil, 100 mg/(kg·d) or 200 mg/(kg·d) TCS, respectively, for 14 d via intragastric administration. TCS was dissolved in corn oil at a concentration of 20 mg/ml or 40 mg/ml. Afterward, the mice were anesthetized, and blood samples were collected for routine testing, and serum and biochemical analyses. The mice were perfused with PBS followed by organ weighing and collection. The liver tissues were dissected to perform scRNA-seq and histological analysis, and the remaining tissues were frozen in liquid nitrogen and stored at − 80 °C.

### Measurement of TCS concentration

The metabolites in serum and liver tissue samples were extracted with methanol. TCS standards were prepared at gradient concentrations of 5, 10, 50, 100, 500 and 1000 ng/ml. All the standards and extracted samples were measured in triplicate by liquid chromatography tandem-mass spectrometry (LC–MS/MS). The areas under the curve of the extracted ion chromatogram of TCS were integrated to represent the level of TCS. The standard curve was plotted with the different concentrations of TCS standards, and TCS concentrations in serum or liver samples were calculated.

### Histological analysis

For histological analysis, a portion of the liver was fixed in 4% paraformaldehyde and embedded in paraffin. After tissues were cut into sections, histological changes were evaluated with hematoxylin and eosin (H&E) staining. For evaluation of the hepatic collagen content and assessment of the degree of fibrosis, the paraffin-embedded liver sections were also stained with Masson’s trichrome and Sirius red.

### Cell culture and treatment

LX-2 (Procell, China) and MIHA (Syngene, China) cells were cultured in DMEM with 10% fetal bovine serum and 1% penicillin/streptomycin in a 5% CO_2_ incubator at 37 °C. For RNA interference experiments, 100 nmol/L siRNA was transfected into cells with Lipofectamine 3000 (Thermo, USA). Cell viability was determined by cell counting kit-8 (CCK-8) reagent according to the manufacturer’s instructions at the appropriate time points as specified in the paper. LX-2 cells were also treated with 10 or 20 μmol/L TCS for 24 h, then subjected to immunological analysis. In addition, BGB324 (MedChemExpress, USA) and recombinant growth arrest specific 6 (rGAS6) protein (MedChemExpress, USA) were used to treat LX-2 cells under the indicated conditions.

### Western blotting

For western blotting, a portion of frozen liver was dissected and ground in liquid nitrogen. The proteins were extracted with RIPA lysis buffer (#P0013B, Beyotime, China) supplemented with 1× Protease Inhibitor Cocktail (#P8849, Sigma, USA). Protein concentrations were determined by Pierce™ BCA Protein Assay Kit (#23225, Thermo, USA). Equal amounts of proteins from different mice liver samples were separated by SDS-PAGE and transferred to PVDF membranes. The membranes were then blocked with 5% skim milk in TBST for 1 h and incubated with primary antibodies overnight at 4 °C. The antibodies included anti-alpha smooth muscle actin (α-SMA) [1:1000; #19245s, Cell Signaling Technology (CST), USA], anti-phospho-Akt (Thr308) (1:1000; #13038S, CST, USA), anti-pan-Akt (1:1000; #4691T, CST, USA) and anti-GAPDH (1:50,000; #60004-1-Ig, Proteintech, China), and corresponding secondary antibody was incubated with membrane for 1 h after washed thrice with TBST. The protein bands were then washed thrice with TBST and visualized with Clarity Western ECL Substrate (#170-5061, Bio-Rad, USA) by using G:BOX Chemi XX9 (Syngene, UK) and semi-quantitative analysis was performed by ImageJ (National Institutes of Health, USA).

### Immunofluorescence

The frozen mouse liver tissue embedded in O.C.T. (#4583, SAKURA, Japan) was cut into 10 μm thick sections. The sections were fixed with acetone/methanol (4:1) for 10 min at − 20 °C, washed with TBS twice and blocked with 4% donkey serum for 1 h at room temperature (RT). Sections were then incubated with the indicated primary antibodies, including rabbit anti-collagen I (1:100; #PAB13488, Abnova, Taiwan, China), rabbit anti-GAS6 (1:200; #13795-1-AP, Proteintech, China), rabbit anti-integrin subunit beta 1 (ITGB1) (1:200; #26918-1-AP, Proteintech, China), rat anti-CD68 (1:100; #14-0681-82, eBioscience, USA), rabbit anti-desmin (DES) (1:200; #16520-1-AP, Proteintech, China) and rabbit anti-lymphatic vessel endothelial hyaluronan receptor 1 (LYVE1) (1:100; #ab281587, Abcam, UK) at RT for 1 h. After being washed with TBST thrice, the sections were then incubated with corresponding Donkey anti-Rat/Rabbit Alexa Fluor 488/594-conjugated secondary antibodies (1:1000; #A-21206-21209, Thermo, USA) for 1 h at RT. Finally, the sections were counterstained and mounted with DAPI, and observed with fluorescence microscopy.

### Preparation of single cell suspension

Samples from the mice from control and TCS-200 groups were subjected to scRNA-seq analysis. Briefly, three liver samples from each group were dissected and cut into small pieces, followed by dissociation using the Liver Dissociation Kit for mouse (Miltenyi Biotec, Germany). The dissociated cell solution was then filtered through a 70-μm cell strainer (Greiner, Germany) and rinsed with 15 ml DMEM. The cells were pelleted by centrifugation at 300 *g* for 10 min, followed by resuspending with 1 ml PBS. Subsequently, 10 ml of 1× Red Blood Cell Lysis Solution (Miltenyi Biotec, Germany) was added to lyse red blood cells. Samples were then washed with PBS twice to obtain single-cell suspensions.

### scRNA-seq and data preprocessing

The single-cell suspensions were used for scRNA-seq library construction with the Single Cell 3′ Reagent Kit v3.1 (10× Genomics) according to the manufacturer’s instructions. The constructed libraries were sequenced on the Illumina HiseqXTEN platform.

The Cell Ranger Software Suite (v6.1.1) was used to perform sample de-multiplexing, barcode processing and single-cell 3′ unique molecular identifier (UMI) counting with the mm10 reference mouse genome obtained from Ensembl. Specifically, Spliced Transcripts Alignment to a Reference (STAR) was used for FASTQ alignment. Cell barcodes were then automatically determined on the basis of the distribution of the UMI count. Finally, the gene-barcode matrix of all six samples was integrated with Seurat (v4.0.3) to remove batch effects. Quality control was first performed independently to identify appropriate thresholds for each individual sample. Gene-barcode matrices for each sample generated by Cell Ranger were loaded into R (https://www.r-project.org/) as a Seurat object for filtering, data normalization, dimension reduction, clustering and differentially expressed gene (DEGs) analysis. Genes were excluded if they were detectable in fewer than three cells. The following criteria were then applied to each cell: gene number between 200 and 5000; UMI count between 500 and 20,000; and mitochondrial gene percentage below 0.15 or 0.25. After filtering, a total of 76,209 cells (37,841 for the control group and 38,368 for TCS-200 group) remained for further analysis.

Next, *SCTransform* was used to normalize each sample and select highly variable genes. To further ensure that clustering would not be influenced by batch effects associated with mouse conditions, we performed the data integration method implemented by Seurat for *SCTransform*-normalized data. Especially, the *PrepSCTIntegration* function was run to identify anchors, and the normalization method parameter was set to the value SCT when running *FindIntegrationAnchors* and *IntegrateData*, to obtain integrated data including six samples.

The integrated gene-barcode matrix was analyzed with PCA by using the top 3000 variable genes, and a shared nearest neighbor graph was constructed on the basis of the Euclidean distance in the low-dimensional subspace spanned by the selected significant principal components (dims = 1:40). Cells were clustered at an appropriate resolution (resolution = 0.1), then visualized with the two-dimensional uniform manifold approximation and projection for dimension reduction (UMAP) algorithm. DEG analysis for each cluster was performed with the Wilcoxon rank-sum test. Cell types were identified with various known markers. For different cell types, cells were grouped on the basis of known markers and analyzed with Seurat in a similar manner.

### Differential gene expression and gene functional enrichment analysis

DEG analysis for each cell type was performed by the Wilcoxon rank-sum test with the Seurat function *FindAllMarkers*. We firstly filtered out the cell types that were either missing or accounted for fewer than 25% of the cells in the comparison groups. Differential expression analysis was performed to generate a set of differential genes (|log_2_ fold change|> 0.25, adjusted *P* value < 0.05). Visualization of markers was performed by violin plots or heatmap using the R packages MySeuratWrappers (v0.1.0) and pheatmap (v1.0.12).

For DEGs, gene ontology (GO) and Kyoto Encyclopedia of Genes and Genomes (KEGG) pathway analyses were performed with the R package clusterProfiler (v4.0.2) [33]. Multiple hypothesis testing correction was performed with the Benjamini–Hochberg procedure. Pathway analysis was performed by R package gene set variation analysis (GSVA) (v1.40.0) [34] with MSigDB Hallmark gene sets, and the differential gene sets were calculated with the R package limma (v3.48.1). Results with adjusted *P* value < 0.05 were further visualized with the R package ggplot2 (v3.3.5).

### Transcription factor (TF) regulatory network analysis

TF regulons were identified with pySCENIC (v0.11.2) [35], a computational method to predict critical regulators and identify cell state from scRNA-seq data. The gene co-expression network was first generated with the gradient boosting machine via the *grn* function. Cis-regulatory motif analysis was performed with pre-computed databases from cisTargetDB via the *ctx* function. The AUCell algorithm was used to score the activity of different regulons via the *aucell* function. To identify the cell-type specific regulons, we calculated the regulon specificity score (RSS) of each cell type via the function *regulon_specificity_scores* and visualized the results with the R package pheatmap. Finally, the specific regulons including TF and target genes were imported into Cytoscape (v3.6.1) [36] for visualization.

### Cell trajectory analysis

Cell trajectory analysis for hepatocytes, hepatic stellate cells (HSCs), B cells and macrophages was performed with the R package monocle (v2.20.0) [37]. DEGs in each subtype were input as variable genes. Then dimensionality reduction was applied to the data with the Reversed Graph Embedding algorithm. Finally, cell ordering was performed with manifold learning via the function *orderCells* and visualized via the function *plot_cell_trajectory*. Furthermore, branches that appeared in the trajectory were analyzed with branched expression analysis modeling (BEAM) to discover DEGs between branches via the function *BEAM* and were visualized via the function *plot_genes_branched_heatmap*.

### RNA velocity-based estimation of cellular transition probability

RNA velocity analysis was performed with velocyto (v0.17) [38] with the default pipeline based on a steady-state model, and the future mRNA abundance of each gene from the ratio of spliced and unspliced mRNA levels was predicted and used as input data for scVelo (v0.2.4) [39]. This procedure enabled estimation of RNA velocity with a dynamic model to learn the full transcriptional dynamics of splicing kinetics. The unspliced/spliced phase trajectory was visualized via the function *velocity_embedding_stream* with UMAP coordinates of endothelial cells (Endos) from the Seurat package as the embedding coordinates for plotting.

### Cell–cell communication inference

Cellular communication analysis was performed with cellphonedb (v2.1.4) [40], on the basis of the ligand-receptor interactions in different cell types. First, the R package biomaRt (v2.48.3) was used to transform gene names from mouse to human. Then the normalized genes expression matrix and cell type meta information served as the input for cellphonedb. The function *method statistical_analysis* was used to calculate the counts of ligand-receptor pairs of different cell types. The function *echartr* in the R package recharts (v0.2-1) was used to perform data visualization. We manually selected ligand-receptor pairs on the basis of *P* value < 0.05 and the mean expression of the average level in the present clusters.

### Label-free proteomics measurement and data analysis

LX-2 and MIHA cells were treated with 10 μmol/L TCS for 24 h. The cells were then collected and the proteins were extracted with 8 mol/L urea in 1% sodium deoxycholate (SDC, w/v) buffer. Proteins were reduced and alkylated with DL-Dithiothreitol (DTT) and Iodoacetamide (IAA). After digesting with trypsin, the resulting peptides were desalted and subsequently analyzed in data-independent acquisition (DIA) mode with LC–MS/MS. The data were analyzed with DIA-NN software. The R package limma (v3.50.3) was used to identify significantly differentially expressed proteins (DEPs) with fold change ≥ 1.2 and adjusted *P* value < 0.05, and the results were visualized with volcano plots and heatmaps. The biological processes enrichment analysis of DEPs was performed with the GO database.

### Metabolomic data analysis

LX-2 and MIHA cells were treated with TCS as described above, and the metabolites were then extracted and analyzed with LC–MS/MS. Raw files were input into Compound Discoverer 3.1 software to perform identification and quantification by matching with the mzCloud, mzVault and MassList databases. MetaboAnalyst 5.0 web server was used to perform the downstream analysis with default parameters. Significantly differentially expressed metabolites (DEMs) were identified on the basis of fold change ≥ 1.2 and *P* value < 0.05.

### Statistical analysis

In non-scRNA-seq and mass spec data sets, data are presented as mean ± standard deviation unless stated otherwise by Graphpad Prism 8.0 (GraphPad Inc.). Schematic diagrams were created with BioRender.com. Two-tailed *t*-test was used to analyze the statistical differences between two groups and ordinary one-way analysis of variance (ANOVA) was used for multiple groups, unless otherwise mentioned. The differential tissue usages (in control and TCS group) were statistically significant based on a chi-square test to evaluate the cell proportion biases of subtypes for each sample [41]. *P* value < 0.05 was considered statistically significant.

## Results

### TCS induces hepatocyte hypertrophy in mice

**Fig. 1:**
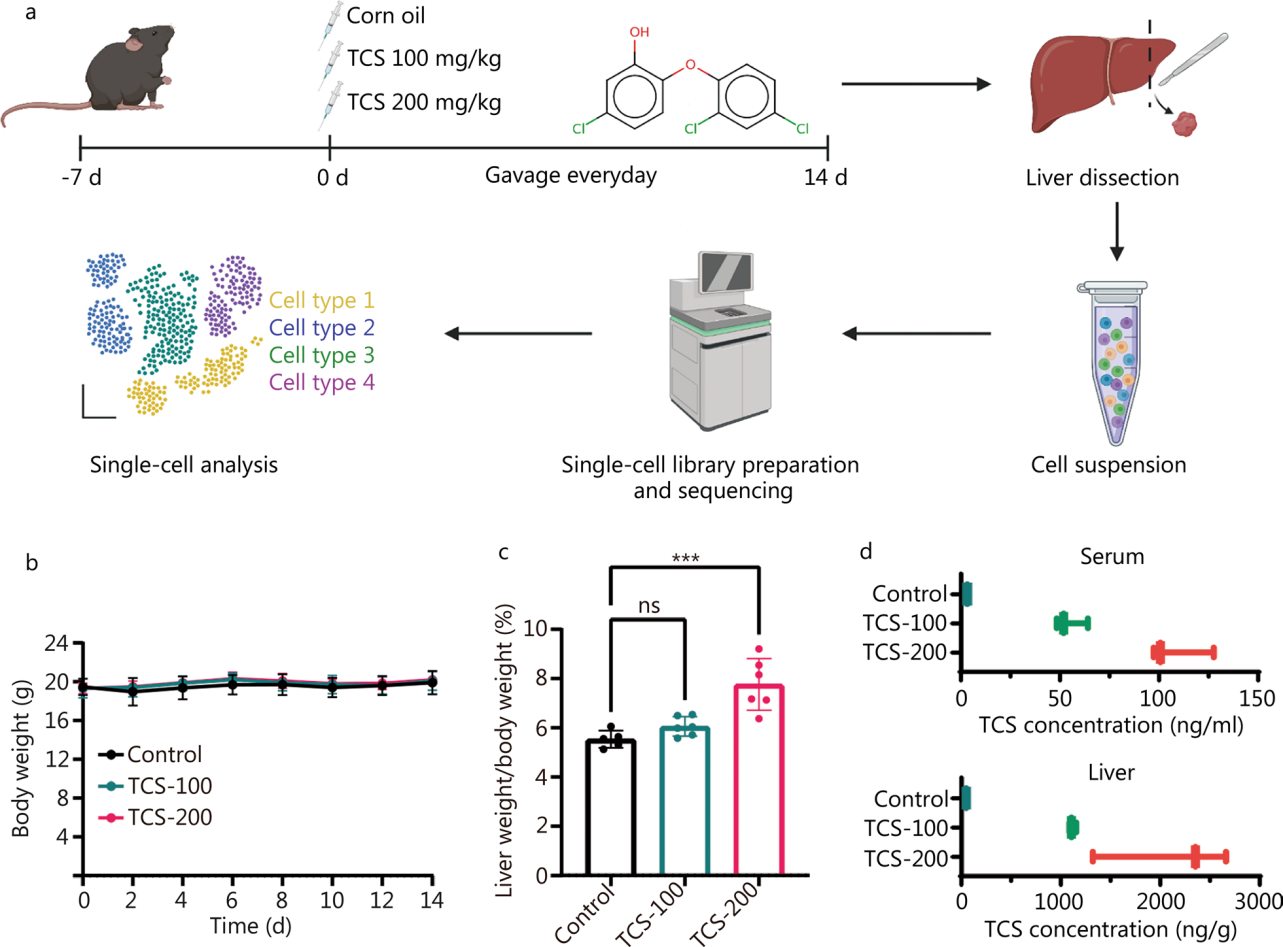
TCS induced hepatocyte hypertrophy in mice liver. **a** Experimental scheme and workflow diagram. **b** Body weight of mice in different groups. **c** The liver/body weight ratio in different groups (*n* = 6). **d** The TCS concentrations in mice serum and liver tissues after 14 d treatment (*n* = 3). TCS triclosan, ns non-significant, ***P < 0.001

We established a relatively short term TCS exposure mouse model to profile the early hepatotoxicity effects of TCS at the single cell level (Fig. 1a). After feeding with TCS for 14 d, the mice did not show any obvious signs of severe toxicity, as indicated by constant body weight (Fig. 1b). In addition, most of the blood or serum biomarkers in the panel were not affected, although some groups showed statistically significant changes with a small effective size (Additional file 1: Fig. S1a, b). The H&E staining on liver tissue showed mild histological changes after TCS exposure (Additional file 1: Fig. S1c). Moreover, the relative liver weights increased in the treatment group, and the difference became statistically significant in mice exposed to 200 mg/kg of TCS (*P* < 0.001, Fig. 1c), in agreement with previous reports [23, 24]. In contrast, the weights of other organs such as the spleen and kidneys were not affected (*P* > 0.05, Additional file 1: Fig. S1d). We then determined the TCS concentrations in the serum and liver tissue with LC–MS/MS (Additional file 1: Fig. S2). The serum concentrations of free TCS were 54.8 ng/ml (0.19 μmol/L) and 108.56 ng/ml (0.37 μmol/L) after exposure to 100 mg/kg and 200 mg/kg of TCS, respectively (Fig. 1d). The concentrations of TCS in the liver were much higher, reaching 1117.51 ng/g and 2112.73 ng/g, respectively (Fig. 1d), suggesting that the liver is the main organ of accumulation and metabolism of TCS in the body [42]. In summary, 14 d of TCS exposure caused obvious hepatocyte hypertrophy at a dose of 200 mg/(kg·d). This group was then chosen for further single-cell analysis to better understand the underlying mechanism.

### Single-cell transcriptome profiling identifies different cell types

**Fig. 2:**
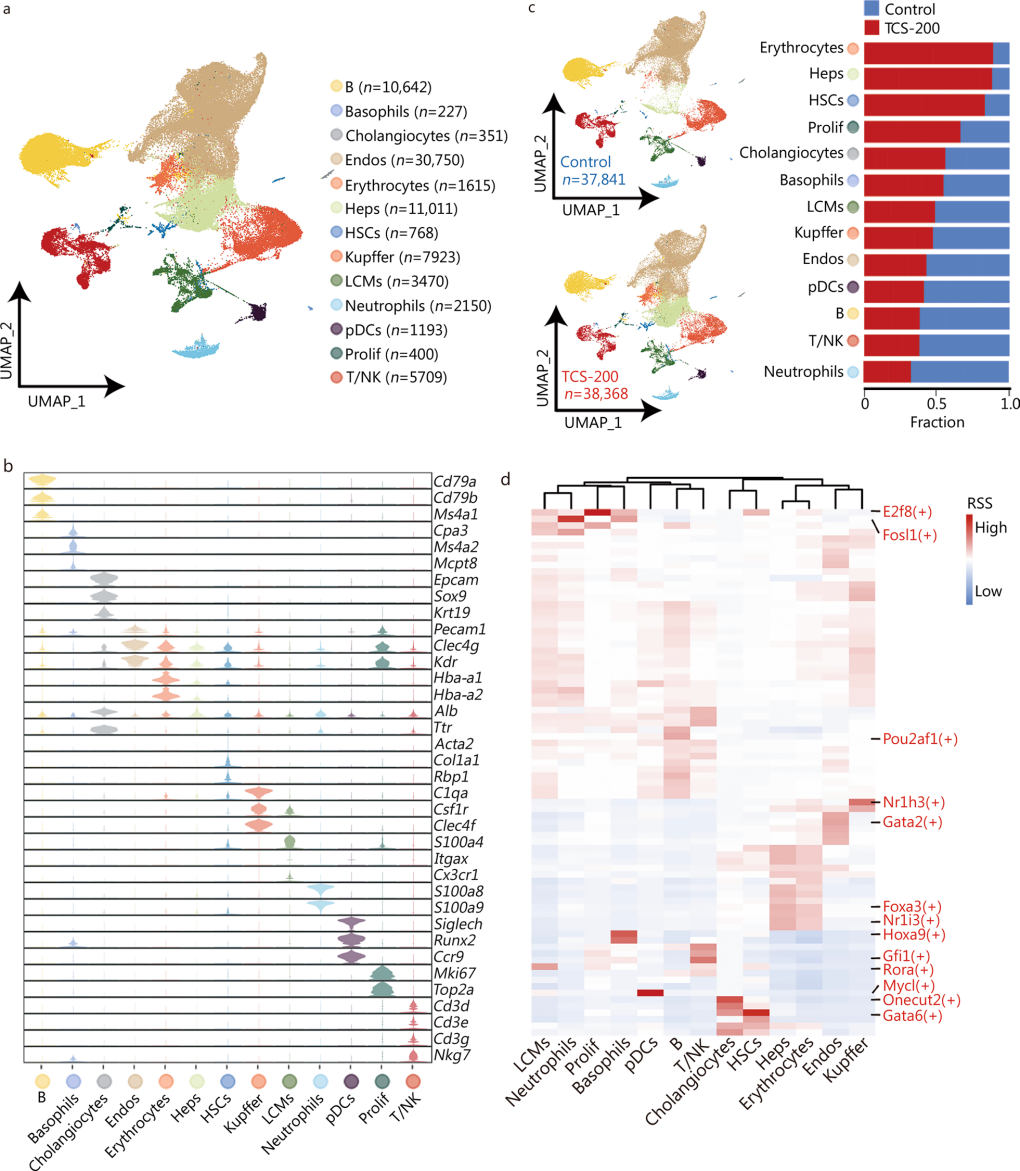
Cell diversity in mice liver cells delineated by single-cell transcriptome. **a** UMAP visualization of 13 cell types based on 76,209 single-cell transcriptomes. Cell counts for each individual cell type are indicated in parentheses. Each dot represents a single cell. **b** Violin plots showing the expression levels of representative markers in each cell type. **c** Distribution of each cell type in control and TCS-200 groups. The bar chart showed the relative fraction of each cell type in different groups. **d** Heatmap of transcription factor (TF) activities in each cell type. UMAP Uniform Manifold Approximation and Projection for Dimension Reduction, Endos endothelial cells, Heps hepatocytes, HSCs hepatic stellate cells, LCMs liver capsular macrophages, pDCs plasmacytoid dendritic cells, Prolif proliferative cells, T/NK T/natural killer cells, TCS triclosan, RSS regulon specificity score

We then investigated the cellular and molecular mechanisms of TCS by carrying out a single cell transcriptional profiling of liver tissues from three control and three TCS-treated (group of 200 mg/kg) mice by using 10× Genomics technology. A total of 76,209 cells (37,841 for control group and 38,368 for TCS-200 group) were retained for further analysis, after data quality control at the gene and cell levels (details in Methods, Additional file 1: Fig. S3). We identified 13 major cell types (Fig. 2a) according to the expression of canonical markers in mouse liver (Fig. 2b, c), including B cells (*n* = 10,642, expressing *Cd79a*, *Cd79b* and *Ms4a1*), Basophils (*n* = 227, expressing *Cap3*, *Ms4a2* and *Mcpt8*), Cholangiocytes (*n* = 351, expressing *Epcam*, *Sox9* and *Krt19*), Endos (*n* = 30,750, expressing *Pecam1*, *Clec4g* and *Kdr*), Erythrocytes (*n* = 1615, expressing *Hba-a1* and *Hba-a2*), hepatocytes (Heps, *n* = 11,011, expressing *Alb* and *Ttr*), HSCs (*n* = 768, expressing *Acta2*, *Col1a1* and *Rbp1*), Kupffer cells (*n* = 7923, expressing *C1qa*, *Csf1r* and *Clec4f*), liver capsular macrophages (LCMs, *n* = 3470, expressing *S100a4*, *Itgax* and *Cx3cr1*), Neutrophils (*n* = 2150, expressing *S100a8* and *S100a9*), plasmacytoid dendritic cells (pDCs, *n* = 1193, expressing *Siglech*, *Runx2* and *Ccr9*), proliferative cells (Prolif, *n* = 400, expressing *Mki67* and *Top2a*) and T/natural killer (NK) cells (*n* = 5709, expressing *Cd3d*, *Cd3e*, *Cd3g* and *Nkg7*). The top 10 DEGs of each cell type are shown in Additional file 1: Fig. S4.

To support our cell type identification, we applied single-cell regulatory network inference and clustering to all cells and identified the TFs with the highest RSS in each cell type (Fig. 2d). For instance, E2f8 (an important modulator of the cell cycle that induce cell proliferation) in Prolif cells, Pou2af1 (a lymphocyte transcriptional coactivator expressed mainly in B cells associated with the formation of germinal centers) in B cells, and Nr1i3 (a constitutive androstane receptor that promotes proliferation of liver cells) in Heps. Among these cell types, the proportion of Heps was higher in the TCS-200 than control group, in agreement with our observation of greater liver weight. In addition, the HSCs, Erythrocytes and Prolif cells also showed higher proportions in TCS-200 group than control group (Fig. 2c). We then comprehensively analyzed how TCS affects specific cell (sub)types in exerting its hepatotoxicity effects.

### Heps exhibit proliferation- and fibrosis-associated characteristics after TCS treatment

**Fig. 3:**
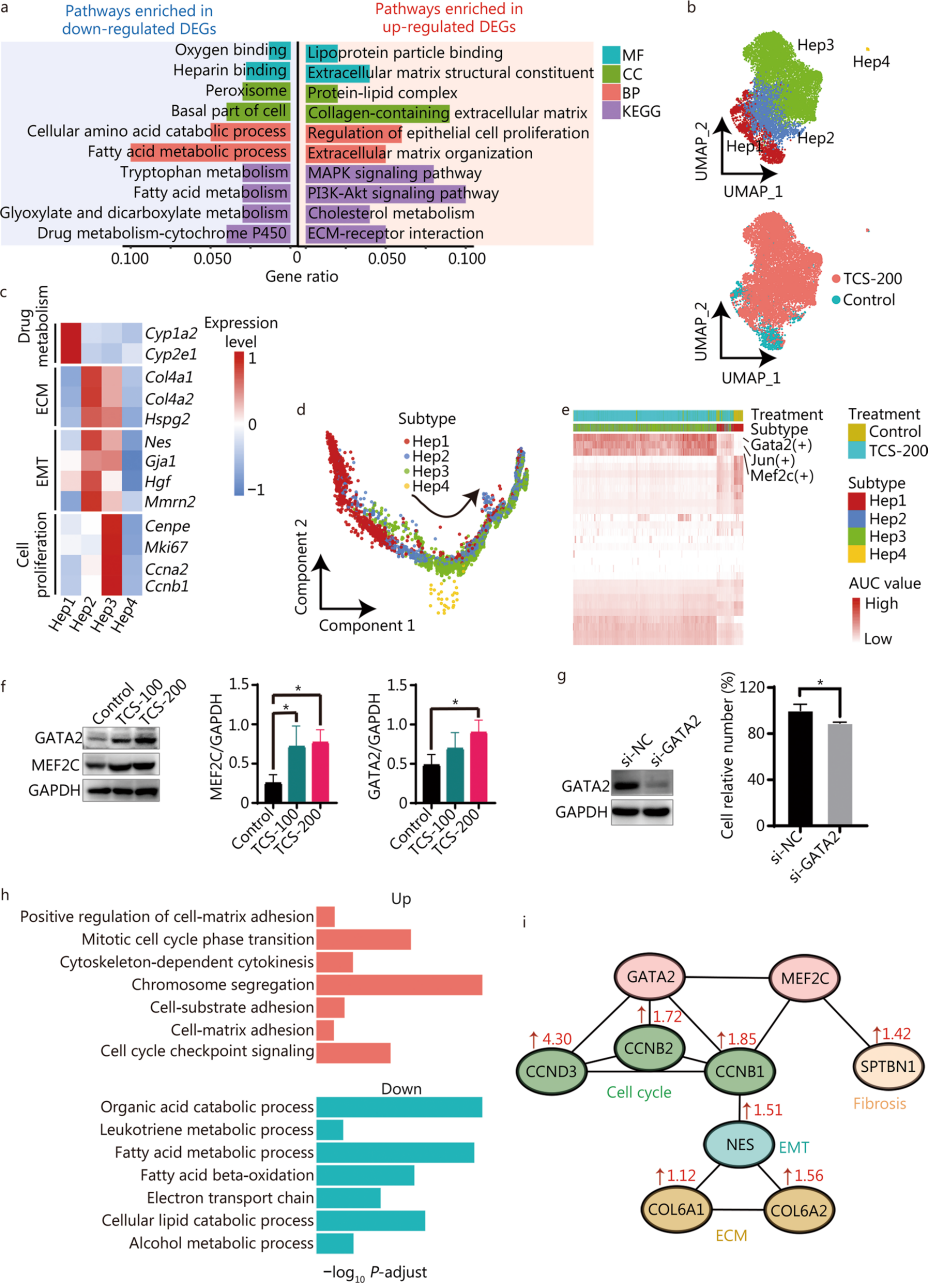
Heterogeneity of hepatocytes (Heps) and their differentiation states. **a** Functional enrichment in the down-regulated (left) and up-regulated (right) DEGs of TCS-200 group vs. control group. **b** UMAP visualization of distinct subtypes of Heps and their distributions in different groups. **c** Heatmap of expression levels of representative markers involved in drug metabolism, ECM, EMT and cell proliferation. **d** Pseudotime trajectory indicating the development of four subtypes, rooting from Hep1. **e** Heatmap showing AUC values of the expression levels of TFs in different subtypes. **f** The protein level of GATA2 and MEF2C in mice liver were detected by Western blotting. **g** Cell viability measured by CCK-8 assay after 72 h after transfection with si-NC for NC (normal control) or si-GATA2. **h** Bar plots showing the biological processes enrich up- and down-regulated DEPs after TCS treatment. **i** The potential regulatory relationship of GATA2 and MEF2C controlled downstream proteins leading to cell proliferation, ECM reorganization and liver fibrosis. Numbers indicate the fold change of protein between TCS-200 and control groups from proteomics dataset. TCS triclosan, DEGs differentially expressed genes, ECM extracellular matrix, MF molecular function, CC cellular component, BP biological process, KEGG Kyoto Encyclopedia of Genes and Genomes, AUC area under the curve, TF transcription factor, UMAP uniform manifold approximation and projection for dimension reduction, EMT epithelial-mesenchymal transition

As described above, the number of Heps significantly increased after TCS treatment (Fig. 2c). To explore how TCS alters Heps, we analyzed the DEGs in the transcriptomes of control and TCS (200 mg/kg)-treated Heps (Additional file 1: Fig. S5a). Compared with those in control group, Heps in TCS-200 group exhibited greater expression of genes involved in extracellular matrix (ECM) structural constituent, ECM organization, collagen-containing ECM, and regulation of epithelial cell proliferation (Fig. 3a). In contrast, the expression of genes involved in fatty acid metabolic process, tryptophan metabolism and drug metabolism were significantly down-regulated, thereby indicating the possible impairment of the metabolic functions of Heps, as further confirmed by metabolomics analysis (Fig. 3a; Additional file 1: Fig. S5b, c). The GSVA analysis results indicated that pathways associated with Notch signaling, epithelial-mesenchymal transition (EMT) and Hedgehog signaling (Additional file 1: Fig. S5d), which are associated with liver regeneration and fibrosis, were also activated in TCS-200 group.

Next, Heps were divided into four subtypes at a higher resolution, which were termed Hep1, Hep2, Hep3 and Hep4, respectively (Fig. 3b). Cells from control group clustered mainly in Hep1, whereas cells in TCS-200 group clustered mainly in Hep2 and Hep3. Clustering analysis of these subtypes revealed unique transcriptomic signatures. Functional enrichment analysis suggested the involvement of Hep1 in retinol and fatty acid metabolism, and the involvement of Hep3 in collagen trimer formation and binding (Additional file 1: Fig. S5e). Interestingly, Hep2 cells were involved in both retinol metabolism and collagen related processes, thus suggesting a mixed or transitional state of Hep1 and Hep3 subtypes. Furthermore, the drug metabolism-associated signatures were enriched in Hep1 cells, ECM- and EMT-associated genes were enriched in Hep2 and Hep3 cells, and cell proliferation signals were intensely enriched in Hep3 cells (Fig. 3c).

To understand the transcriptional dynamics of Heps during TCS treatment, we reconstructed cell–cell relationships through pseudotime trajectory analysis (Fig. 3d). The results suggested that the trajectory began with Hep1 cells, and most Hep2 cells progressed to the Hep3 phenotype, in agreement with the UMAP location and pathway enrichment analysis (Fig. 3b, Additional file 1: Fig. S5e).

In addition, we used pySCENIC to map the gene regulatory networks governing these subtypes. Marked differences in the regulon activity were observed among different treatment and cell subtypes, thus again supporting that distinct cell states were induced by TCS (Fig. 3e). The Hep2 and Hep3 cells in TCS-200 group had elevated regulon activity for Gata2 and Mef2c (Additional file 1: Fig. S5f), which are known to be associated with development and oncogenesis [43, 44]. The up-regulation of the protein expression levels of GATA2 and MEF2C was also confirmed through both in vivo and in vitro treatment experiments (Fig. 3f, g; Additional file 1: Fig. S5g). Target genes regulated by Gata2 and Mef2c are enriched in processes associated with the response to transforming growth factor beta (*Tgfb2*, *Eng* and *Bmp2*), EMT (*Tgfb2*, *Bmp2*, *Eng* and *Gja1*) and ECM organization (*Timp3, Sparc, Tgfb2*, *Ramp2*, *Adamts1*, *Serpinh1*, *Eng* and *Bmp2*) and collagen fiber assembly (*Bgn*) (Additional file 1: Fig. S5h). Moreover, Jun, an important TF regulating a large number of genes, was up-regulated by TCS treatment. Jun promotes hepatocyte survival and the progression from steatosis to NASH, whereas the expression of Jun in non-parenchymal liver cells is known to correlate with fibrosis [45].

We also performed quantitative proteomics analysis of DEPs after TCS treatment of the MIHA normal human hepatocyte cell line (Additional file 1: Fig. S5i). The up-regulated DEPs were enriched mainly in cell–matrix adhesion and the cell cycle, whereas the down-regulated DEPs were enriched in metabolic processes (Fig. 3h). We examined the measured changes in expression of the proteins regulated by *Gata2* and *Mef2c*, including those associated with drug metabolism (CYP4F12, CYP27A1 and CYP4F11), cell cycle (CENPF, CCND3, CCNB1 and CCNB2) and collagen formation (COL6A1, COL6A2 and NES), all of which showed trends consistent with the scRNA-seq results (Additional file 1: Fig. S5j). Thus, we next used the Search Tool for the Retrieval of INteracting Genes/proteins (STRING) database to construct a protein–protein interaction network for these proteins, to better understand the regulatory relationships among these key DEPs (Fig. 3i). GATA2 plays an important role in controlling the expression of cell cycle associated proteins (such as CCNB1, CCNB2 and CCND3). GATA2 and MEF2C up-regulate the expression of CCNB1 and NES (involved in EMT), thereby up-regulating the collagen proteins COL6A1 and COL6A2. Furthermore, MEF2C directly activates SPTBN1 and consequently induces liver fibrosis [46].

In summary, these findings indicated that TCS induces the proliferation of normal Heps, down-regulates retinol and fatty acid metabolism, and promotes fibrogenesis by accelerating ECM-associated processes, in which Gata2 and Mef2c are the key driving TFs.

### HSCs are activated after TCS treatment

HSCs, one of the key non-parenchymal cell types in the liver, serve as the major origin of ECM proteins after injury. Beyond the increase in HSCs in TCS-200 group (Fig. 2c), GO analysis indicated that TCS induced the down-regulation of genes involved in lipid oxidation and storage, and the up-regulation of genes involved in collagen biosynthetic process, macrophage chemotaxis and the cell cycle (Additional file 1: Fig. S6a).

**Fig. 4:**
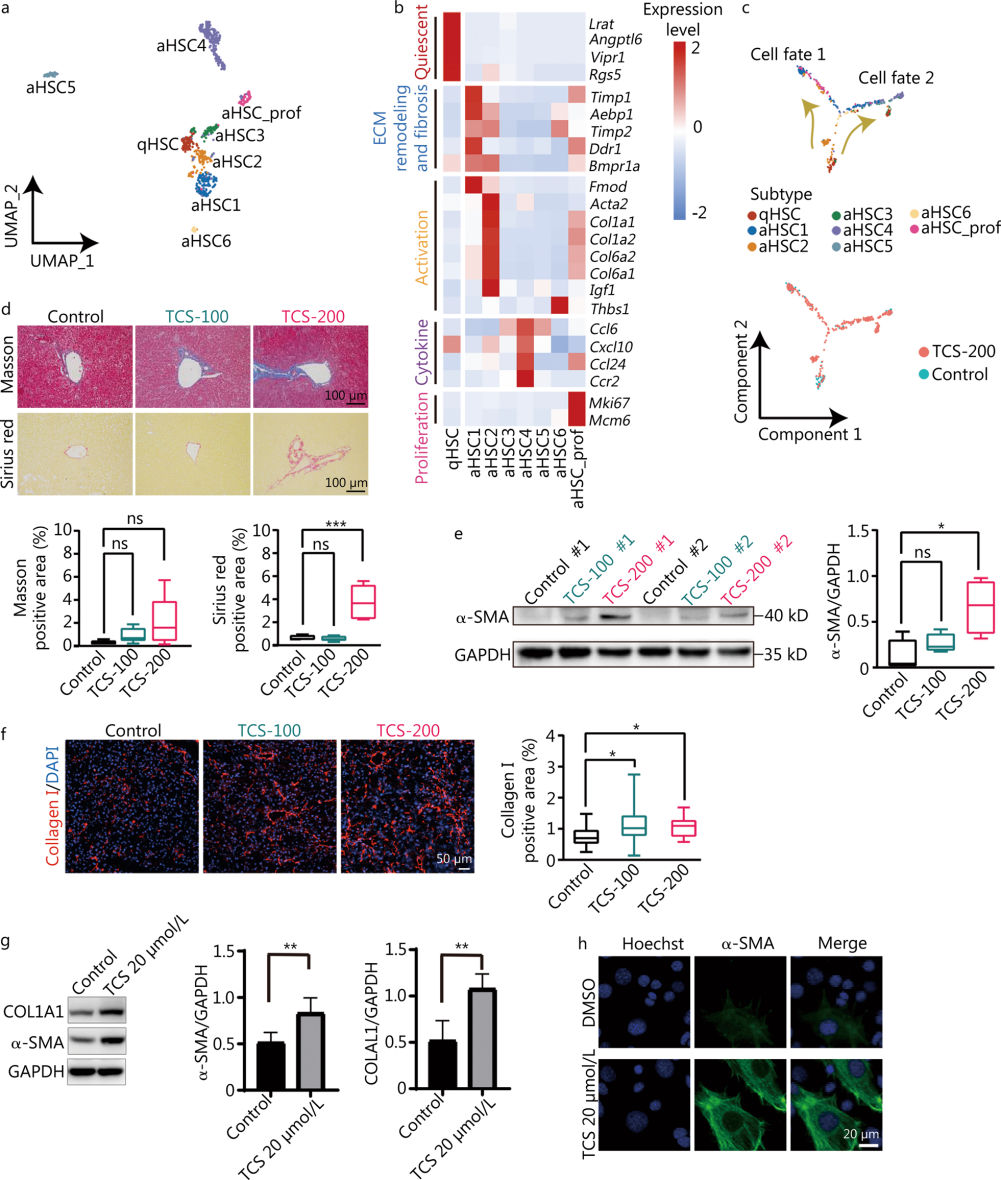
Transcriptomic roadmap of HSCs activation. **a** UMAP visualization of eight distinct subtypes of HSCs. **b** Heatmap of expression levels of representative markers related to cell quiescence, ECM remodeling and fibrosis, activation, cytokine and proliferation. **c** Pseudotime trajectory indicating the development of HSCs subtypes. Cell fate 1 represents trajectory from qHSC to aHSC1, cell fate 2 represents from qHSC to aHSC4. **d** Masson and Sirius red staining and the corresponding quantification of Masson- and Sirius red-positive areas (*n* = 5). Scale bar = 1000 μm. **e** Western blotting analysis of α-SMA expression levels in mice liver with or without TCS treatment, and the corresponding quantification of α-SMA protein expression levels, relative to GADPH loading control (*n* = 4). **f** Immunofluorescence staining of collagen I on mouse liver frozen sections (*n* = 4). The collagen I positive area was analyzed by ImageJ. Scale bar = 50 μm. **g** The protein expression of COL1A1 and α-SMA in LX-2 cells after TCS treatment. **h** Immunofluorescence staining of α-SMA in LX-2 cells after TCS or DMSO treatment. Scale bar = 20 μm. HSCs hepatic stellate cells, UMAP uniform manifold approximation and projection for dimension reduction, qHSC quiescent hepatic stellate cells, aHSC activated hepatic stellate cells, ECM extracellular matrix, TCS triclosan, α-SMA alpha smooth muscle actin, ns non-significant; *P < 0.05, **P < 0.01

HSCs were divided into one quiescent subtype (qHSC) and seven activated subtypes (aHSC1–6 and aHSC_prof), among which aHSC_prof exhibited cell proliferation features, at a higher resolution (Fig. 4a). Among these subtypes, qHSC accounted for the highest proportion in control sample (32.3%, *n* = 42 of 130) but only 7.2% (*n* = 46 of 638) in the TCS treatment sample (Additional file 1: Fig. S6b). Notably, aHSC1 and aHSC2 showed overexpression of genes involved in ECM remodeling and fibrosis, thus suggesting roles in the development of liver fibrosis. In addition, aHSC4 was enriched in the expression of chemotactic cytokines including *Ccl6*, *Cxcl10*, *Ccl24* and *Ccr2* (Fig. 4b).

Similarly, pseudotime analysis was performed to follow the transcriptomic changes in HSCs after TCS treatment. The results indicated that qHSC developed into primarily aHSC1 (referred to as cell fate 1) or aHSC4 (referred to as cell fate 2) (Fig. 4c), accompanied by aHSC2, aHSC_prof and so on. To further explore the molecular events underlying the different trajectories, we used BEAM to analyze the branch-dependent DEGs (Additional file 1: Fig. S6c). Cell fate 2, the lineage from qHSC to aHSC4, showed the overexpression of genes involved in chemotaxis such as *Ccl6*, *Cxcl2*, *Cxcl13*, *Ccl3*, *Ccr2* and *Ccr12*, in agreement with the subtype cell markers shown in Fig. 4b. In contrast, cell fate 1, the lineage from qHSC to aHSC1, was enriched in the expression of genes involved in ECM organization and collagen fibril organization.

Because the activation of HSCs is known to be the key driver of liver fibrogenesis, and our analysis indicated that TCS induced the proliferation and activation of HSCs, we performed Masson’s trichrome and Sirius red staining and western blotting to validate the progression of fibrogenesis. TCS was found to increase collagen deposition in mouse liver and to have a statistically significant effect in the 200 mg/kg dosage group (Fig. 4d). In addition, the protein expression level of α-SMA (an important marker of fibrogenesis) (Fig. 4e) and the hepatic deposition level of collagen I were elevated, as expected (Fig. 4f). We also validated that TCS promoted the expression of α-SMA and collagen I in LX-2, a human HSC line (Fig. 4g, h).

In the proteomics analysis performed on LX-2 cells, up-regulated DEPs were mostly associated with cell cycle transition, whereas down-regulated DEPs were enriched in RNA localization, regulation of glycogen biosynthetic process and oxidative phosphorylation (Additional file 1: Fig. S6d, e), in good agreement with the scRNA-seq results (Additional file 1: Fig. S6a). Notably, proteins associated with collagen organization (COL6A2, COL4A2, COL6A1, COL16A1 and BMP1) and the cell cycle (UBE2E2, TOP2A and CDK14) were elevated in TCS-200 group (Additional file 1: Fig. S6f), thus further supporting the proliferation and activation characterization of HSCs after TCS treatment.

These results revealed that TCS treatment drives the development of HSCs from quiescent cells into several different activated phenotypes, particularly the aHSC1 subtype, which showed overexpression of ECM genes (directly associated with fibrosis), and the aHSC4 subtype, which showed overexpression of chemotactic chemokines (associated with cell migration).

### Endos undergo dysfunction and capillarization after TCS treatment

Liver Endos, including sinusoidal endothelial cells (LSECs) and vascular endothelial cells (LVECs), as well as lymphatic endothelial cells, are the largest group of cells within the non-parenchymal cells of the liver, and they regulate liver homeostasis and intrahepatic vascular tone [47]. The genes up-regulated after TCS treatment among Endos, with respect to the expression in control group, were found to be involved in cellular response to external stimulus, the canonical Wnt signaling pathway, response to hypoxia and fibroblast proliferation (Additional file 1: Fig. S7), whereas the down-regulated genes were involved in the regulation of vasculature development, endothelial cell proliferation and chemotaxis.

**Fig. 5:**
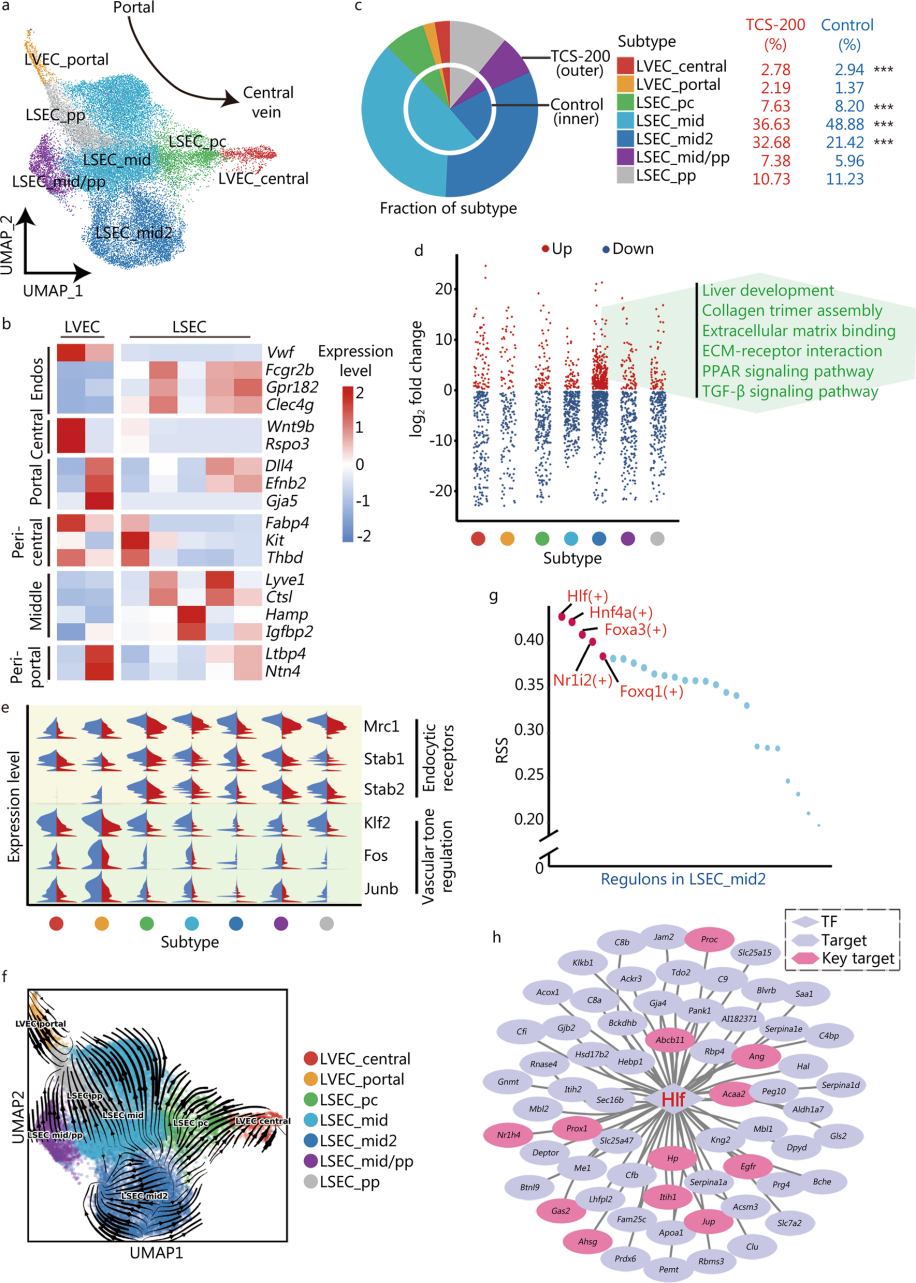
Distinct endothelial cells according to the spatial locations. **a** UMAP visualization of the 7 distinct subtypes of Endos based on spatial distribution. **b** Heatmap of the expression levels of representative markers indicating generalized Endos as well as central, pericentral, middle, periportal and portal position. **c** Pie chart showing the fraction of each endothelial subtype. **d** Strip chart showing DEGs of each subtype after TCS-200 treatment. The texts in green color represents the pathways enriched in the up-regulated DEGs in LSEC_mid2. **e** Split violin plot of the expression levels of genes associated with endocytic receptors and vascular tone regulation. **f** RNA velocity analysis of different subtypes of Endos, indicating LSEC capillarization. **g** Scatter diagram showing RSS of TFs in LSEC_mid2 subtype. The top 5 TFs ordered by scores were listed. **h** Transcription regulatory network constructed by Hlf and its target genes. Red text represents TFs, black text for targets (target), pink hexagon for genes related to ECM, tissue remodeling and response to hypoxia (key target). Endos endothelial cells, LVEC liver vascular endothelial cells, LSEC liver sinusoidal endothelial cells, UMAP uniform manifold approximation and projection for dimension reduction, DEGs differentially expressed genes, TCS triclosan, ECM extracellular matrix, RSS regulon specificity score, TFs transcription factors; ***P < 0.001

According to the spatial lobular locations, we divided Endos into two LVEC subtypes and five LSEC subtypes (Fig. 5a), which were mapped on the liver lobule from the portal tract to the central venous regions according to the expression levels of portal, periportal, middle, pericentral and central markers (Fig. 5b). Notably, the proportion of the LSEC_mid2 subtype exhibited the most significant increase after TCS treatment, and contained more DEGs than control group (Fig. 5c, d). The functional enrichment of the up-regulated genes in this subtype suggested their participation in liver development, collagen trimer assembly, ECM binding, PPAR signaling pathway and TGF-β signaling pathway (Fig. 5d), which are associated with the promotion of liver fibrosis. We then examined TCS-induced physiological functional changes in Endos. Endos remove circulating antigens and toxins via different endocytic receptors [48], including Mrc1, Stab1 and Stab2, all of which significantly decreased after TCS treatment (Fig. 5e). Moreover, Endos respond to increased shear stress to maintain normal vascular through the activation of TFs such as Klf2, Fos and Junb, which were markedly suppressed, thus suggesting TCS-induced dysfunction of Endos.

At the onset of liver fibrosis, LSECs undergo capillarization (loss of fenestrae) and produce basement membrane—a phenotype mimicking that of common LVECs [47–49]. To explore whether this phenotypic change also existed in our model, we performed RNA velocity analysis to estimate the cellular transition probability and indeed found a tendency for most LSEC_pc and some LSEC_mid2 cells to transform into LVEC_central cells (Fig. 5f). Furthermore, we constructed gene regulatory networks for the seven subtypes and found that Hlf exhibited the highest RSS in LSEC_mid2 cells (Fig. 5g). The target genes regulated by Hlf were mainly involved in liver development (*Proc*, *Abcb11*, *Prox1*, *Egfr* and *Hp*), extracellular structure organization (*Gas2*, *Itih1*, *Jup* and *Nr1h4*), tissue remodeling (*Ahsg* and *Egfr*) and response to hypoxia (*Ang*, *Acaa2*, *Hp* and *Egfr*) (Fig. 5h).

Overall, these results indicated TCS-induced dysfunction of Endos, showing diminished endocytic and vascular developmental/regulatory ability, whereas the ECM-associated processes and collagen trimer assembly were up-regulated. In addition, the phenotypic shift of most LSEC_pc and some LSEC_mid2 toward LVEC_central cells (i.e., capillarization), indicating the occurrence of liver fibrosis, appeared to be under the control of Hlf activation.

### B plasma cells with fibrotic characteristics increase after TCS treatment

**Fig. 6:**
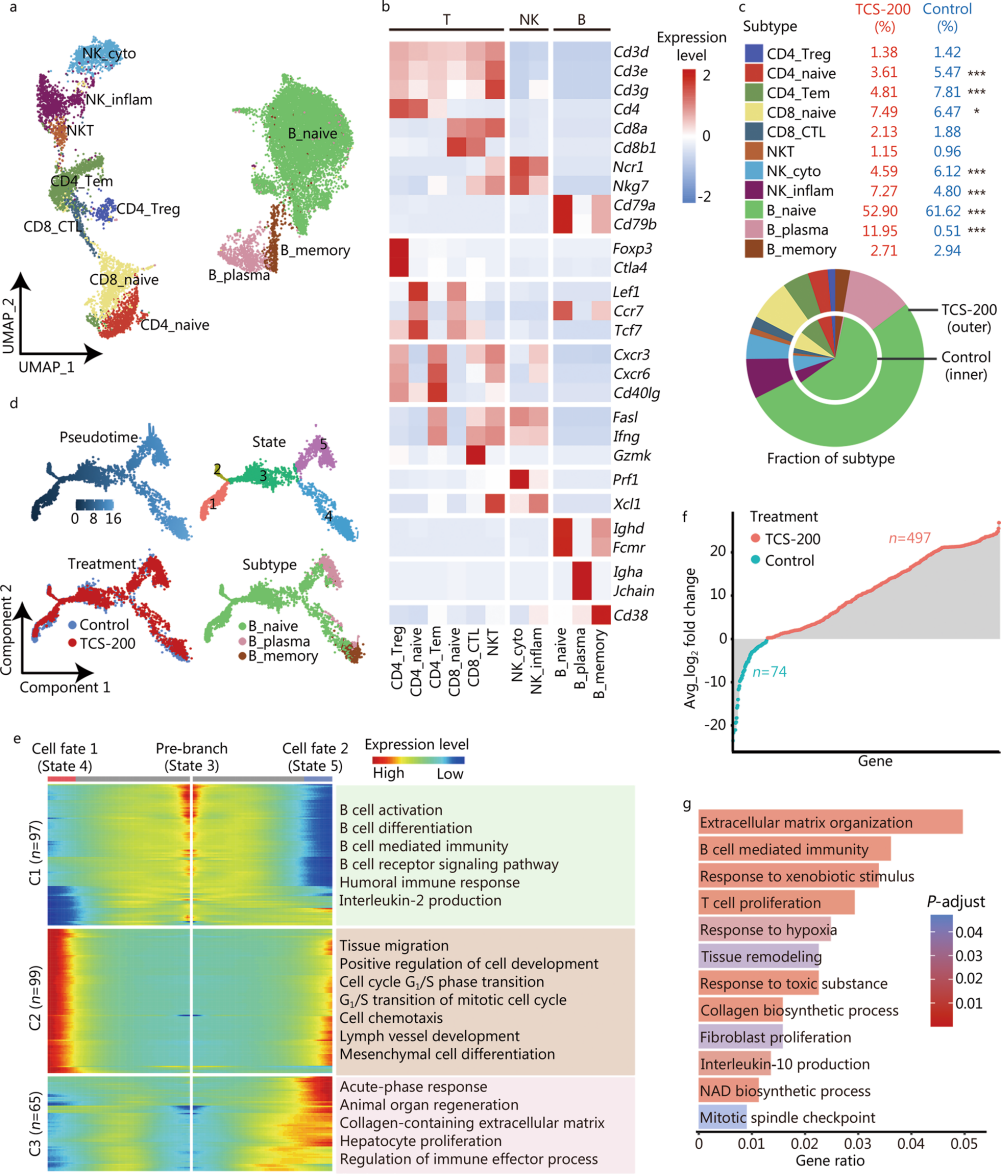
Dynamic regulation of lymphocytes after TCS treatment. **a** UMAP visualization of distinct subtypes of lymphocytes including B, T and NK cells. **b** Heatmap of the expression levels of representative markers indicating lymphocytes, regulatory, naive, memory, effector, cytotoxic, and inflammatory functions. **c** Pie chart showing the relative fraction of each lymphocyte subtype. **d** Pseudotime trajectory indicating the development of B cell subtypes. The different color schemes represent the segregation based on pseudotime, cell state, treatment and subtype, respectively. **e** BEAM showing genes involved in the differential development of cell fate 1 (State 4) and cell fate 2 (State 5), and the enriched GO terms were listed at right. **f** Lollipop chart depicting the DEGs of B plasma cells after the TCS-200 treatment. Red represents up-regulated and blue represents down-regulated genes. **g** Bar graph showing the functions enriched in up-regulated DEGs of B plasma cells after the TCS-200 treatment. Length represents gene ratio and color represents adjusted *P* value. UMAP uniform manifold approximation and projection for dimension reduction, NK natural killer cells, C1 cluster 1, C2 cluster 2, C3 cluster 3, BEAM branched expression analysis modeling, GO Gene Ontology, DEGs differentially expressed genes, TCS triclosan; *P < 0.05, ***P < 0.001

Lymphocytes, including T, NK and B cells, play important roles in the pathogenesis of liver disease. We next analyzed how TCS affects lymphocytes in mouse livers. First, we identified the subtypes of lymphocytes according to the expression of key marker proteins. NK cells were divided into two subtypes: NK_cyto (expressing *Prf1*) and NK_inflam (expressing *Xcl1*), which have cytotoxic and inflammatory characteristics, respectively (Fig. 6a, b). Similarly, T cells contained naive (expressing *Lef1*, *Ccr7* and *Tcf7*), effector (expressing *Fasl*, *Ifng* and *Gzmk*), memory (expressing *Cxcr3*, *Cxcr6* and *Cd40lg*) and regulatory (expressing *Foxp3* and *Ctla4*) subtypes, and NKT cells were characterized by both T and NK cell signatures. Three subtypes of B cells were identified: naive (expressing *Ighd*, *Fcmr*), memory (expressing *Cd38*) and plasma (acting as effector B cells with overexpression of *Igha* and *Jchain*) cells. Notably, B_plasma showed the most significant increase (up to 23-fold) after TCS-200 treatment (11.95% vs. 0.51%) (Fig. 6c), thus supporting the requirement of B cells for liver fibrogenesis [50].

Consequently, we reconstructed the developmental trajectory of B cells (Fig. 6d) and found that B_naive cells differentiated into B_plasma or B_memory subtypes, the latter of which was also accompanied by a minor population of B_plasma cells. To further explore the molecular mechanisms underlying the different trajectories, we retrieved the branch-dependent DEGs and performed biological processes enrichment analysis (Fig. 6e). The results suggested that B_naive cells (State 3) were enriched in genes involved in B cell activation and differentiation; B_memory cells (cell fate 1, State 4) were enriched in genes involved in tissue migration and the cell cycle transition; and B_plasma cells (cell fate 2, State 5) were enriched in genes involved in acute-phase response, collagen-containing ECM and hepatocyte proliferation. Furthermore, DEGs of B_plasma cells after TCS treatment (up = 497, down = 74) were identified (Fig. 6f). The up-regulated genes were enriched in ECM organization, response to hypoxia, tissue remodeling and fibroblast proliferation (Fig. 6g), many of which are associated with liver fibrogenesis.

These results revealed that the TCS-induced increase in B plasma cells might be the key subtype of lymphocytes contributing to the progression of liver fibrosis.

### LCMs and Kupffer cells display M2-skewed phenotype after TCS treatment

Myeloid cells, consisting of granulocytes, monocytes, macrophages, dendritic cells and so on, show heterogeneous distributions and self-replenish in response to drug-induced injury in the liver [51]. LCMs and Kupffer cells can be further divided into M1 (expressing *Cd86* and *Cd68*), M2 (expressing *Tgfb2*, *Wnt5a* and *Mrc1*) and proliferation (expressing *Mki67*, *Stmn1*, *Cenpf* and *Ccnb2*) subtypes (Additional file 1: Fig. S8a, b). Among them, the proportions of the M2 and proliferation subtypes increased after TCS treatment (Additional file 1: Fig. S8c). Interestingly, the pseudotime trajectory analysis also suggested differentiation of the M1 subtype of LCM and Kupffer cells into the M2 subtype, along with the distribution of the proliferation cells (Additional file 1: Fig. S8d), thus resulting in M2-skewed polarization. Previous study has demonstrated that macrophages in the fibrotic liver exhibit M2-preponderant activation [52], thereby further supporting TCS treatment-induced liver fibrosis.

Moreover, after TCS treatment, 376 (up = 239, down = 137) and 1118 (up = 703, down = 415) DEGs were identified in the LCM_M2 and KC_M2 subtypes, respectively (Additional file 1: Fig. S8e), including the up-regulation of several pro-fibrosis genes, such as *Ccl2* and *Il1b* in LCM_M2, *Il6* and *Mmp2* in KC_M2, as well as *Apom* and *Spp1* in both LCM_M2 and KC_M2. In addition, genes associated with ECM (*Fbn1*, *Col14a1*, *Ecm1*, *Mgp* and *Col1a1*), ECM fibrosis (*Pdgfra* and *Lrp1*), ECM remodeling (*Adamts5*) and proteoglycan assembly (*Lum*) were up-regulated in KC_M2 cells. Finally, we performed functional enrichment analysis of the up-regulated DEGs of KC_M2 (Additional file 1: Fig. S8f), which indicated enrichment in biological progresses involved in collagen ECM organization, fibroblast proliferation, macrophage chemotaxis, response to hypoxia and T-helper (Th)2 cell cytokine production. Of note, the excessive secretion of Th2 cytokine has been reported to be associated with excessive activation of M2 macrophages and to be positively correlated with the severity of fibrosis [53].

In summary, these results suggested the skewing of macrophages from M1 to M2 phenotype, and the discovery of several key genes likely to contribute to the progression of liver fibrosis.

### Cell–cell communication crosstalk within the crucial subtypes

To explore differential cell–cell interactions after TCS treatment, we constructed a cellular communication network among different cell types with potential ligand-receptor pairs (Additional file 1: Fig. S9a). We observed relatively more counts of interactions in HSCs, Kupffer, LCMs and Endos cells acting either as source or target cells. We confirmed the enhanced interactions between macrophages and HSCs, and between macrophages and LSECs, in the TCS treatment group (Additional file 1: Fig. S9b). Given the analytic results for individual cell types, we focused on the cellular communication among the Hep2, Hep3, aHSC1, LSEC_mid2, B_plasma, KC_M2 and LCM_M2 subtypes (Additional file 1: Fig. S9c), all of which showed potential associations with the progression of liver fibrosis after TCS treatment. The interaction counts were particularly high in aHSC1, KC_M2 and LSEC_mid2 cells.

**Fig. 7:**
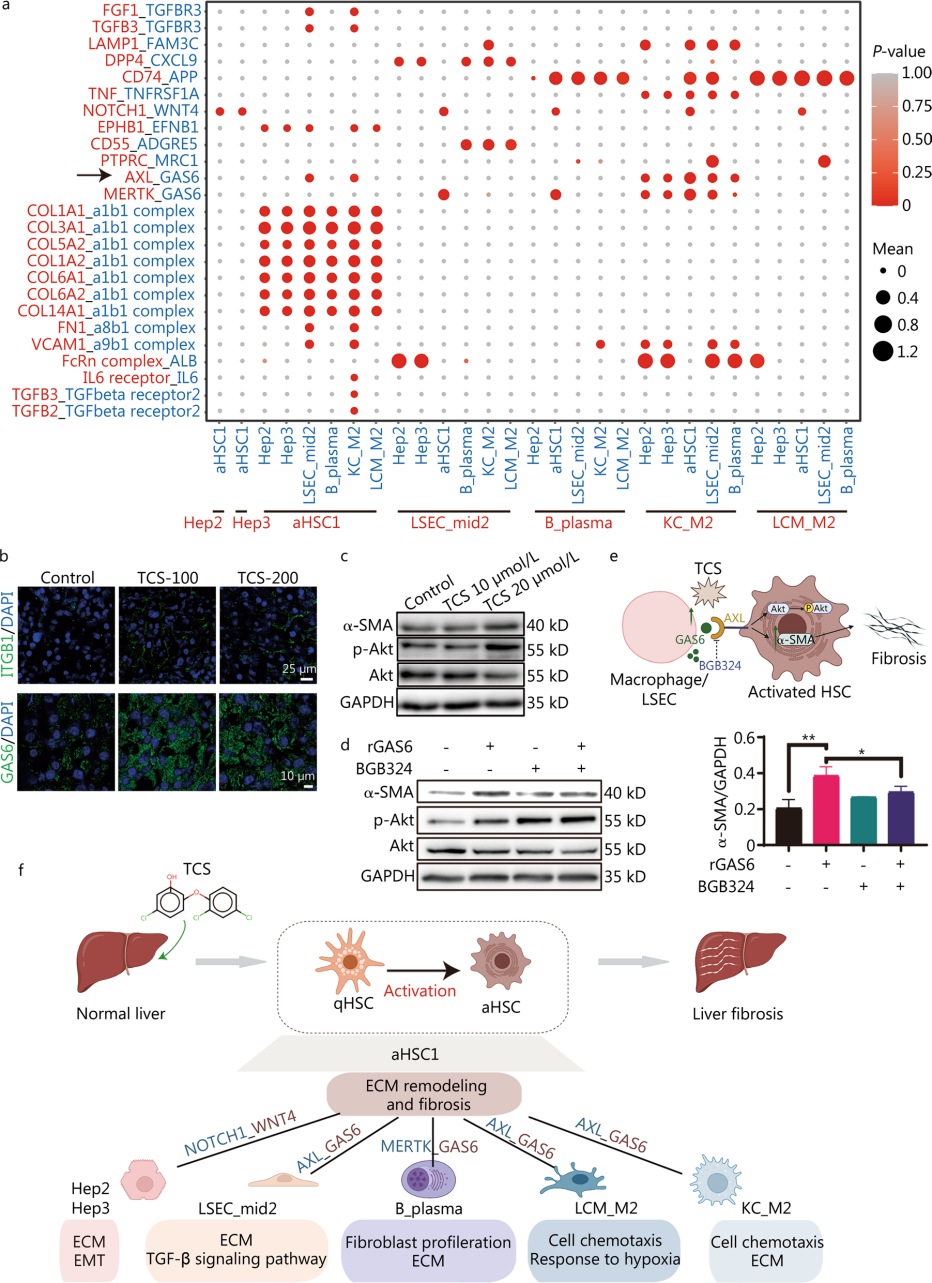
Cell–cell communication crosstalk of different cell types. **a** Dot plot depicting ligand-receptor pairs within different subtypes. Circle sizes indicate mean expression of pairs and colors indicated enrichment of *P*-values in the two subtypes. **b** Immunofluorescence staining of ITGB1 (top) and GAS6 (bottom) on mouse liver frozen sections. Scale bar = 25 μm (top) and scale bar = 10 μm (bottom). **c** LX-2 cells were treated with indicated concentration of TCS for 24 h, and the expression of α-SMA, p-Akt and total Akt were examined with Western blotting, GAPDH was used as loading control. **d** LX-2 cells were incubated with 500 ng/ml rGAS6 for 30 min and/or pre-incubated with BGB324 (1 μmol/L, 30 min), and the expression of α-SMA, p-Akt and total Akt were examined with Western blotting, GAPDH was used as loading control. **e** Scheme showing the potential fibrosis-related mechanism caused by the combination of GAS6 and AXL in HSCs. **f** Summary and inference of cellular communication induced by TCS on mice liver. ITGB1 integrin beta 1, TCS triclosan, α-SMA alpha smooth muscle actin, p-Akt phosphorylated Akt, rGAS6 recombinant GAS6, HSCs hepatic stellate cells, LSEC liver sinusoidal endothelial cells, qHSC quiescent hepatic stellate cells, aHSC activated hepatic stellate cells, ECM extracellular matrix, EMT epithelial-mesenchymal transition; *P < 0.05, **P < 0.01

The key ligand-receptor pairs forming the interaction network are summarized in Fig. 7a. Many of the ligand-receptor pairs have been reported to be involved in fibrosis, such as IL6 receptor_IL6, TGFB2/3_TGFbeta receptor2/3, NOTCH1_WNT4, MERTK_GAS6 and TNF_TNFRSF1A [54–57]. As shown in Additional file 1: Fig. S9c, aHSC1 exhibited ligand-receptor pairs interacting with the other subtypes. For the ligands on aHSC1 cells, the most significantly enriched pairs comprised collagen molecule and the corresponding complex on all subtypes, in line with excessive ECM formation when HSCs are activated. We confirmed that the protein levels of collagen I and ITGB1 (one subunit of a1b1 complex) were both elevated in TCS-treated mouse livers (Figs. 4f, 7b). Among these ligand-receptor pairs, we observed that GAS6 from LSECs and macrophages showed strong communication with aHSC1 through AXL. It was reported that GAS6_AXL pathway is associated with fibrosis [58], we further verified the involvement of the GAS6_AXL pathway in TCS-induced fibrosis. We first confirmed that the protein level of GAS6 was elevated in TCS-treated mouse liver (Fig. 7b). TCS also promoted the expression of α-SMA and phosphorylated Akt (p-Akt) (Fig. 7c). After incubation with recombinant GAS6 (rGAS6), HSC LX-2 cells also showed increased expression of α-SMA and p-Akt (Fig. 7d). Moreover, the AXL inhibitor BGB324 partially blocked the activation of HSC by rGAS6 (Fig. 7d). Therefore, we experimentally confirmed the potential contributing role of the GAS6_AXL pair in TCS-induced liver fibrosis, thus leading to increased expression of α-SMA (Fig. 7e).

Overall, our study identified several critical ligand-receptor pairs between the selected crucial subtypes, which are known or have been reported to facilitate liver fibrogenesis. Combing the above results, we delineated the key underlying mechanisms of TCS-induced liver fibrosis (Fig. 7f), which were attributable to cell type-specific modulation on Heps (particularly Hep2 and Hep3), Endos (particularly LSEC_mid2), macrophages (particularly KC_M2 and LCM_M2), B cells (particularly B_plasma) and HSCs (particularly aHSC1).

## Discussion

TCS was once considered a safe additive because the median lethal dose (LD_50_) for inducing acute toxicity is as high as 4350 mg/kg body weight in mice and > 5000 mg/kg body weight in dogs [59]. The TCS content in commercial products may reach as high as 17 mmol/L (0.5%) [5]. Although the use of TCS in antiseptic soaps was phased out in the United States in 2016 by FDA, it is still widely used in a wide range of personal care and health care products (such as toothpaste, mouthwash, shampoo, deodorant and cosmetics) in many countries [60–63]. The wide use and chemical persistence of TCS have also led to substantial accumulation in the environment, thus causing severe pollution problems [64, 65].

The toxicity of TCS has been studied in different mouse models with varying dosages or exposure terms. For instance, Wang et al. [23] have demonstrated that TCS induces mouse liver tumors through the chimeric antigen receptor (CAR) and PPARα activation at doses of 0, 10, 100 and 200 mg/kg for 14 or 28 d. Cao et al. [42] have examined dose–response genotoxicity of TCS in mice at doses of 0 to 1000 mg/(kg·d) for 19 d. In the present study, we treated mice with 200 mg/(kg·d) body weight for 14 d by oral gavage, and we provide the first detailed single cell atlas in mouse liver with a focus on the TCS-induced liver fibrosis. Of note, although the dosage used in our study was higher than the typical concentrations in consumer products for daily use, the measured TCS serum concentration was largely within the range of reported human body burden levels (i.e., sub μmol/L in serum), and the mice showed no changes in body weight, or in a panel of blood or serum biomarkers. Nevertheless, liver hypertrophy and pathology were observed after the 14-day treatment, thus confirming prior findings indicating that the liver is the main target organ of TCS [11]. Our study exploited scRNA-seq to illustrate TCS treatment-induced modulation of gene expression in major cell types including Heps, HSCs, Endos, lymphocytes and myeloid cells, and their communication crosstalk. More importantly, the molecular and cellular events underlying TCS-induced liver fibrogenesis were dissected at the single cell level.

TCS has been reported to induce an increase in hepatocyte DNA synthesis and cell proliferation. In our study, Heps in the TCS treatment group exhibited obvious proliferation, mainly driven by Hep2 and Hep3 subtypes. In addition, elevated collagen accumulation was observed after TCS treatment, as verified with Masson’s trichrome and Sirius red staining, as well as immunoblotting and immunofluorescence experiments. The analysis of subtypes suggested their transition from metabolic processes toward proliferation and fibrosis. The Hep2 and Hep3 subtypes were enriched in ECM and EMT related processes, which might be regulated by TFs such as Gata2 and Mef2c.

Activated HSCs are known as the main ECM-producing cells. The transition from quiescence led to two different trajectories: one subtype participated in cell migration, and the other participated in ECM organization. Recently, Zhang et al. [66] have discovered two major trajectories during HSC activation in liver fibrogenesis, from proliferation to either an inflammatory cluster or collagen organization cluster. Notably, we extended the trajectory starting from quiescent HSCs and accompanied by the activated proliferation subtypes.

Other cells, including Endos and immune cells, also regulate the ECM and contribute to the progression of liver fibrosis. LSECs are specialized Endos that form hepatic sinusoids, which lack a basement membrane, thus allowing for solute exchange between the sinusoid lumen and the space of Disse [67]. In the normal liver, Endos remove circulating antigens and toxins via endocytic receptors, and respond to increased shear stress to maintain normal vascular conditions. Our study suggested that TCS treatment induced disorder in endocytosis and dysfunction in vascular regulation, which are the representative features of endothelial dysfunction observed in cirrhosis [68]. During the progression of liver fibrosis, LSEC fenestrae are diminished, thus preventing the exchange in substances and oxygen between the liver parenchyma and sinusoidal blood, which is also known as capillarization, thereby leading to a failure in suppressing HSCs [69]. In our study, LSECs exhibited a phenotypic shift to LVECs, in line with the capillarization features. Furthermore, LSEC_mid2 cells were charactered by middle-zone markers and overexpression of genes associated with ECM and TGF-β signaling pathway. This might be regulated by TF Hlf, which facilitates liver fibrosis through activation of HSCs driven by the HLF/IL-6/STAT3 feedforward circuit [70].

Studies have shown that B cells are required for liver fibrogenesis in an antibody- and T cell-independent manner [71]. Our study revealed that the pro-fibrogenesis function of B cells appeared to be mainly related to the plasma cell, which sharply increased after TCS treatment. The relationship between plasma cells and stellate cells in autoimmune hepatitis has been established, exhibiting co-localization and positive correlation [72]. In agreement with this relationship, genes associated with ECM organization, response to hypoxia and tissue remodeling were up-regulated in plasma cells in our data.

Among myeloid cells, macrophages, including LCM and Kupffer cells, displayed M2-skewed phenotype. M2 macrophages release anti-inflammatory or pro-resolving mediators, which influence wound repair, tissue remodeling and fibrosis [73]. Several up-regulated genes facilitating liver fibrosis were identified in our data. For example, *Il1b* is a potent inflammatory cytokine produced mainly by macrophages that participate in toxic-, ethanol- and NASH-induced fibrosis [74, 75], and it has been found to prolong the survival of HSCs [76]. *Il6* is a key pro-inflammatory and profibrogenic cytokine that drives liver fibrosis [57]. *Mmp2* belongs to the matrix metalloproteinase family, and its increased expression has been found to promote ECM deposition [77]. Additionally, *Ppara*, a nuclear receptor participating in the development of hepatic steatosis induced by TCS [21], was up-regulated after TCS treatment in LSEC_mid2, Hep3, aHSC1 and KC_M2 cells in our study.

Finally, cell–cell communication crosstalk analysis confirmed the central role of HSCs in association with other cell types. Notably, aHSC1 cells exhibited the most interactions with other subtypes when acting as either source or target in ligand-receptor pairing. The combination of GAS6 and AXL was verified to increase the expression of α-SMA and promote fibrosis, collagen molecules and the corresponding complex, IL6 receptor_IL6, TGFB2/3_TGFbeta receptor2/3, MERTK_GAS6 and TNF_TNFRSF1A, in line with the role of aHSC1 as a major executor of fibrogenesis.

## Conclusions

The present study provides the first comprehensive analysis of cellular and biological processes involved in TCS-induced hepatotoxicity through scRNA-seq. Our analysis uncovered the molecular changes in the six main liver cell types, and enabled the construction of an interaction network of cells centering on the activation of HSCs. Our study suggests that TCS modulates different cell types in concert to activate HSCs, promoting fibrogenesis and resulting in hepatotoxicity.

## Supplementary Information

**Additional file 1:** **Fig. S1.** TCS showed no signs of severe toxicity. **Fig. S2.** The XIC (**a**) and mass spectrogram (**b**) of TCS detected in LC–MS/MS. **Fig. S3.** Data distribution after quality control (feature number between 200 and 5000; UMI count between 500 and 20,000; and mitochondrial gene percentage below 0.15 or 0.25) in each sample, and a total of 76,209 cells (37,841 for the control group and 38,368 for TCS-200 group) remained for further analysis. **Fig. S4.** Heatmap showed top 10 DEGs of each cell type based on the average fold change value. **Fig. S5.** Cellular change of hepatocytes (Heps). **Fig. S6.** Cellular change of HSCs. **Fig. S7.** Functions/processes enriched in down-regulated (left) and up-regulated (right) DEGs after TCS treatment in Endos. **Fig. S8.** Emergence of diverse macrophages after TCS treatment. **Fig. S9.** Cell–cell communication change after TCS treatment.

## Data Availability

All the sequencing data are deposited in Genome Sequence Archive (GSA) (https://bigd.big.ac.cn/gsa/) with the accession number of CRA005439.

## Abbreviations

aHSC: Activated hepatic stellate cells
AUC: Area under the curve
BEAM: Branched expression analysis modeling
CAR: Chimeric antigen receptor
CCK-8: Cell counting kit-8
DEGs: Differentially expressed genes
DEMs: Differentially expressed metabolites
DEPs: Differentially expressed proteins
DES: Desmin
DIA: Data-independent acquisition
DTT: DL-Dithiothreitol
ECM: Extracellular matrix
EMT: Epithelial-mesenchymal transition
Endos: Endothelial cells
FDA: Food and Drug Administration
FDR: False discovery rate
GAS6: Growth arrest specific 6
GO: Gene ontology
GSVA: Gene set variation analysis
H&E: Hematoxylin and eosin
Heps: Hepatocytes
HSCs: Hepatic stellate cells
IAA: Iodoacetamide
ITGB1: Integrin subunit beta 1
KEGG: Kyoto Encyclopedia of Genes and Genomes
LC–MS/MS: Liquid chromatography tandem–mass spectrometry
LCMs: Liver capsular macrophages
LD_50_: Median lethal dose
LSECs: Liver sinusoidal endothelial cells
LVECs: Liver vascular endothelial cells
LYVE1: Lymphatic vessel endothelial hyaluronan receptor 1
NASH: Nonalcoholic steatohepatitis
NK: Natural killer cells
p-Akt: Phosphorylated Akt
pDCs: Plasmacytoid dendritic cells
PLS-DA: Partial least squares discriminant analysis
PPARα: Peroxisome proliferator activated receptor α
Prolif: Proliferative cells
qHSC: Quiescent hepatic stellate cells
rGAS6: Recombinant GAS6
RSS: Regulon specificity score
RT: Room temperature
scRNA-seq: Single cell RNA sequencing
SDC: Sodium deoxycholate
STAR: Spliced Transcripts Alignment to a Reference
TCS: Triclosan
TF: Transcription factor
UMAP: Uniform manifold approximation and projection for dimension reduction
UMI: Unique molecular identifier
α-SMA: Alpha smooth muscle actin
