# Supplementary material for: Single-cell transcriptomic dissection of the cellular and molecular events underlying the triclosan-induced liver fibrosis in mice
Source: Mil Med Res. 2023 Feb 22;10:7. doi: 10.1186/s40779-023-00441-3 (PMC9945401; doi:10.1186/s40779-023-00441-3)
Supplement: Supplementary file 1 — Additional file 1: Fig. S1. TCS showed no signs of severe toxicity. Fig. S2. The XIC (a) and mass spectrogram (b) of TCS detected in LC–MS/MS. Fig. S3. Data distribution after quality control (feature number between 200 and 5000; UMI count between 500 and 20,000; and mitochondrial gene percentage below 0.15 or 0.25) in each sample, and a total of 76,209 cells (37,841 for the control group and 38,368 for TCS-200 group) remained for further analysis. Fig. S4. Heatmap showed top 10 DEGs of each cell type based on the average fold change value. Fig. S5. Cellular change of hepatocytes (Heps). Fig. S6. Cellular change of HSCs. Fig. S7. Functions/processes enriched in down-regulated (left) and up-regulated (right) DEGs after TCS treatment in Endos. Fig. S8. Emergence of diverse macrophages after TCS treatment. Fig. S9. Cell–cell communication change after TCS treatment. [file 40779_2023_441_MOESM1_ESM.pdf]

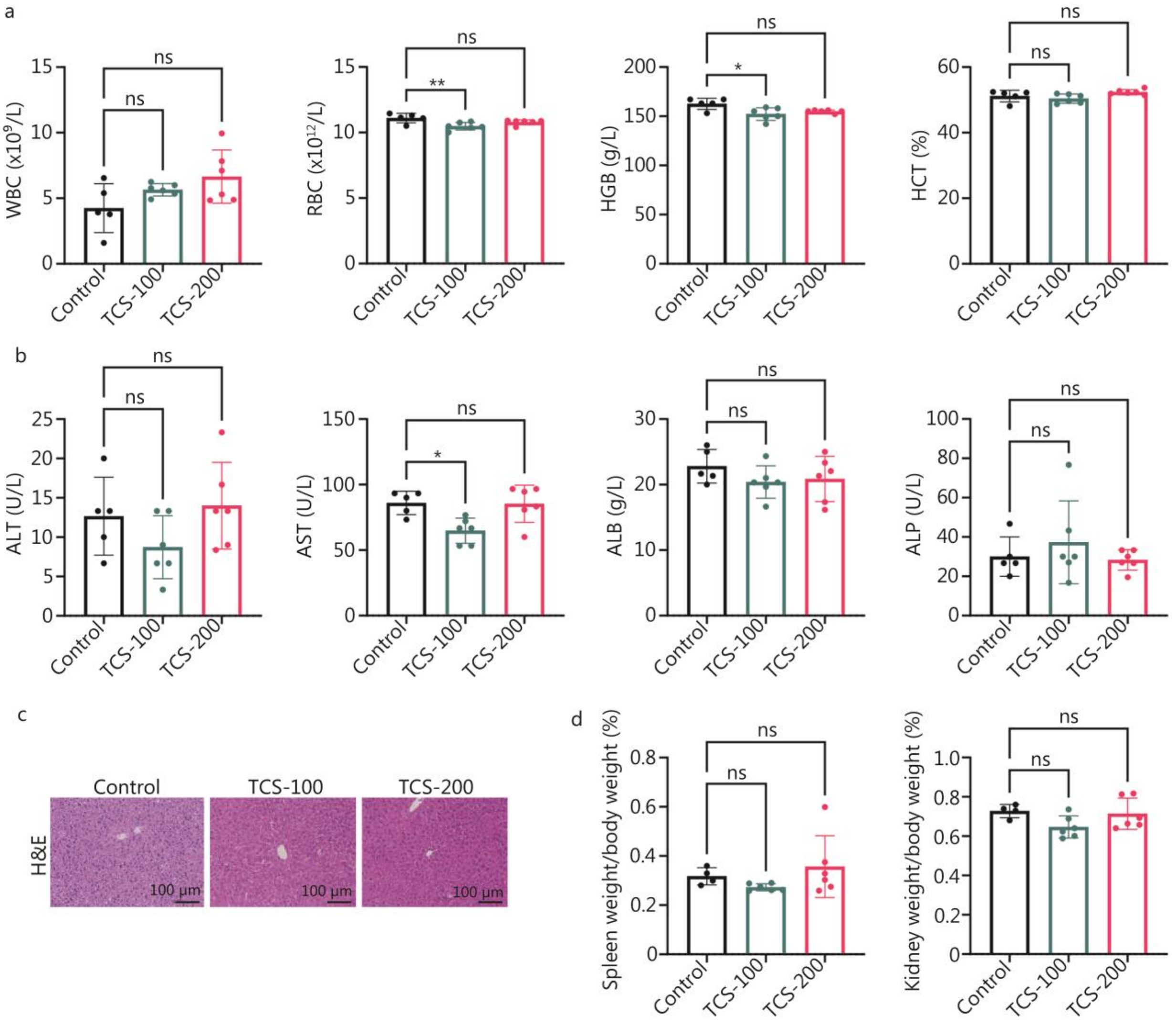

Fig. S1 TCS showed no signs of severe toxicity. **a** Whole blood routine test results of white blood cell (WBC), red blood cell (RBC), hemoglobin (HGB) and hematocrit (HCT). **b** Serum biochemical test results of alanine aminotransferase (ALT), aspartate aminotransferase (AST), albumin (ALB) and alkaline phosphatase (ALP). **c** H&E staining of mice liver treated with or without TCS. Scale bar = 100 μm. **d** Organ/body weight ratios of spleen and kidney between different group mice. TCS triclosan, ns non-significant; $^{*}P < 0.05$, $^{**}P < 0.01$

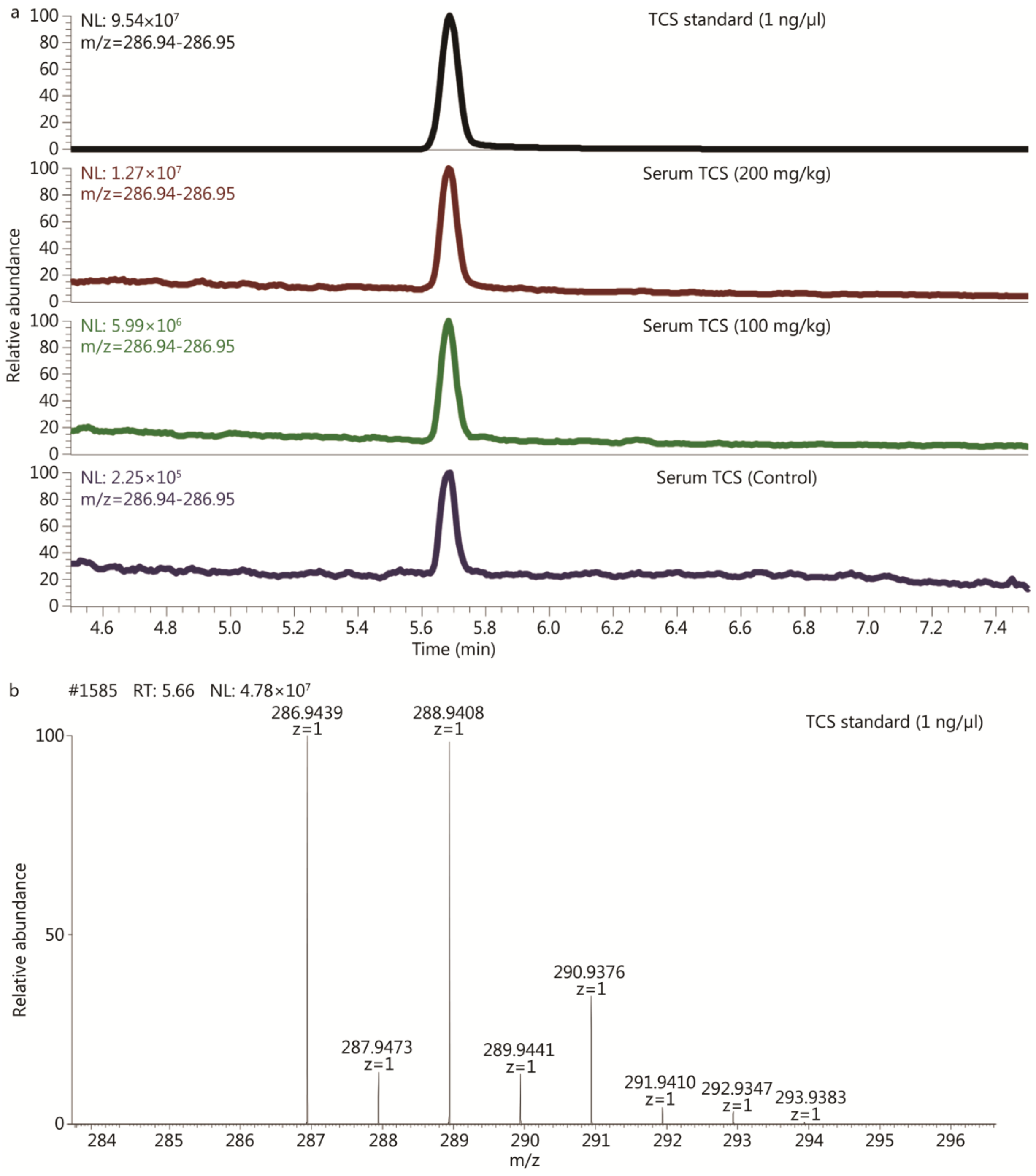

Fig. S2 The XIC (**a**) and mass spectrogram (**b**) of TCS detected in LC-MS/MS. XIC extracted ion chromatogram, TCS triclosan, LC-MS/MS liquid chromatography tandem-mass spectrometry

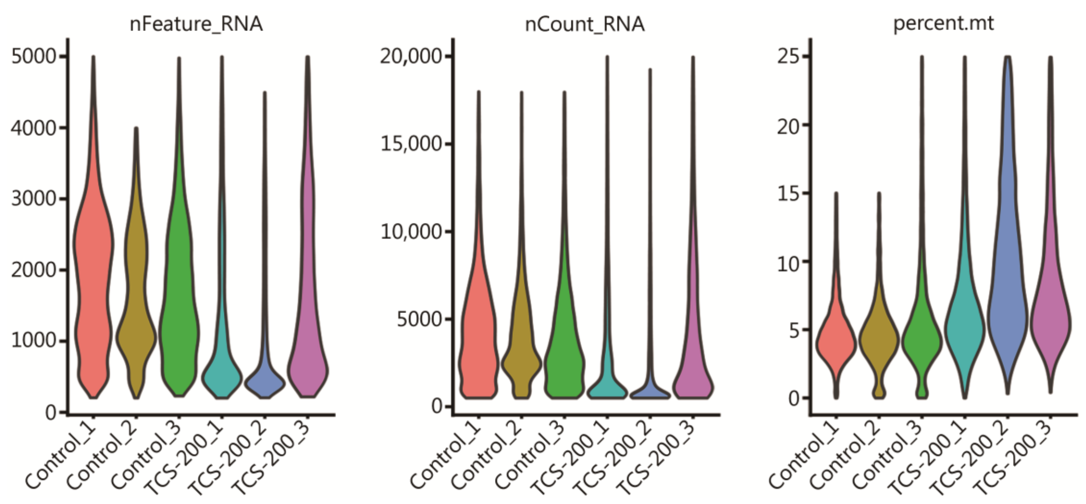

Fig. S3 Data distribution after quality control (feature number between 200 and 5000; UMI count between 500 and 20,000; and mitochondrial gene percentage below 0.15 or 0.25) in each sample, and a total of 76,209 cells (37,841 for the control group and 38,368 for TCS-200 group) remained for further analysis.

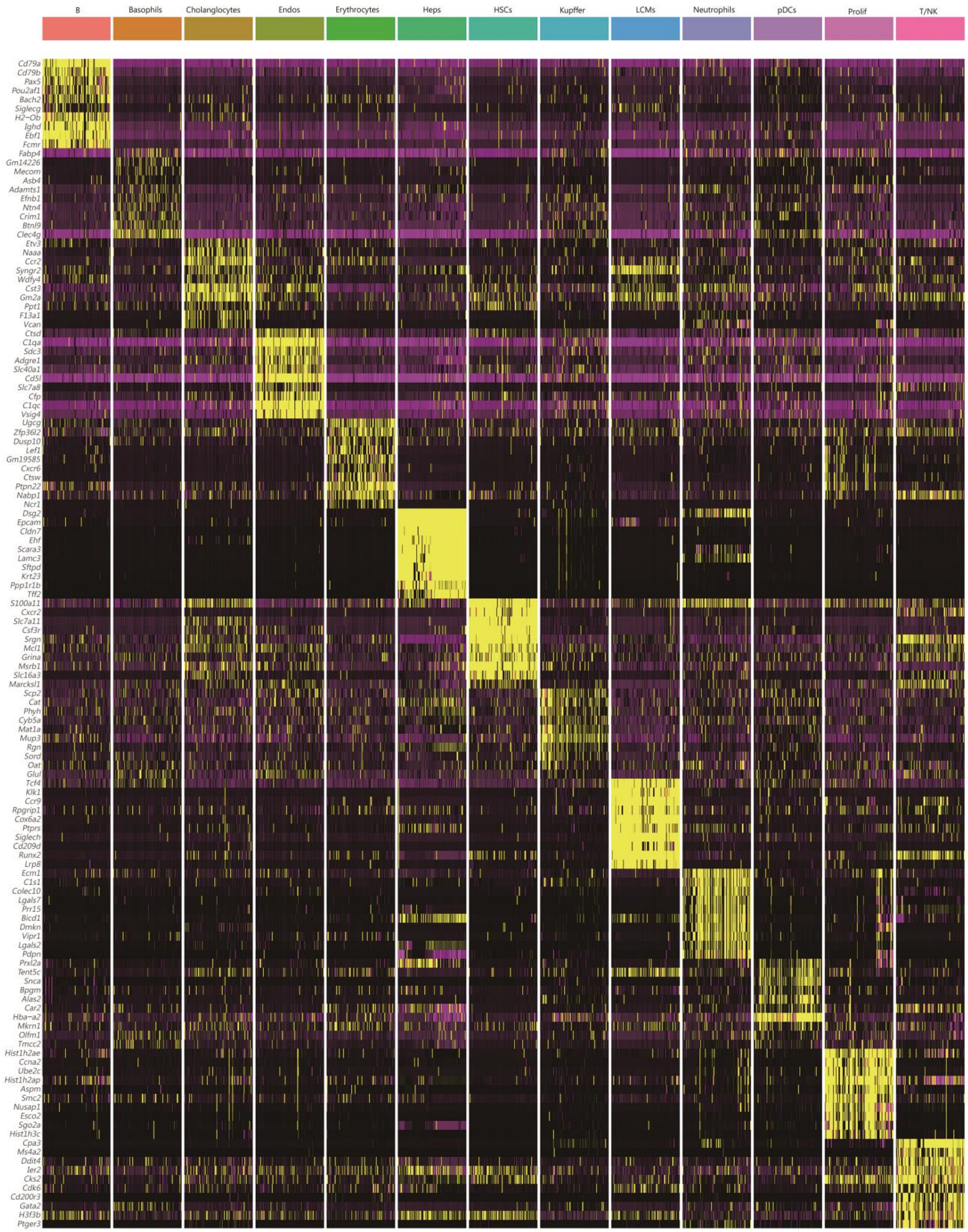

Fig. S4 Heatmap showed top 10 DEGs of each cell type based on the average fold change value. The number of cells was set to downsample = 100. DEGs differentially expressed genes, Endos endothelial cells, Heps hepatocytes, HSCs hepatic stellate cells, LCMs liver capsular macrophages, pDCs plasmacytoid dendritic cells, Prolif proliferative cells, T/NK T/natural killer cells

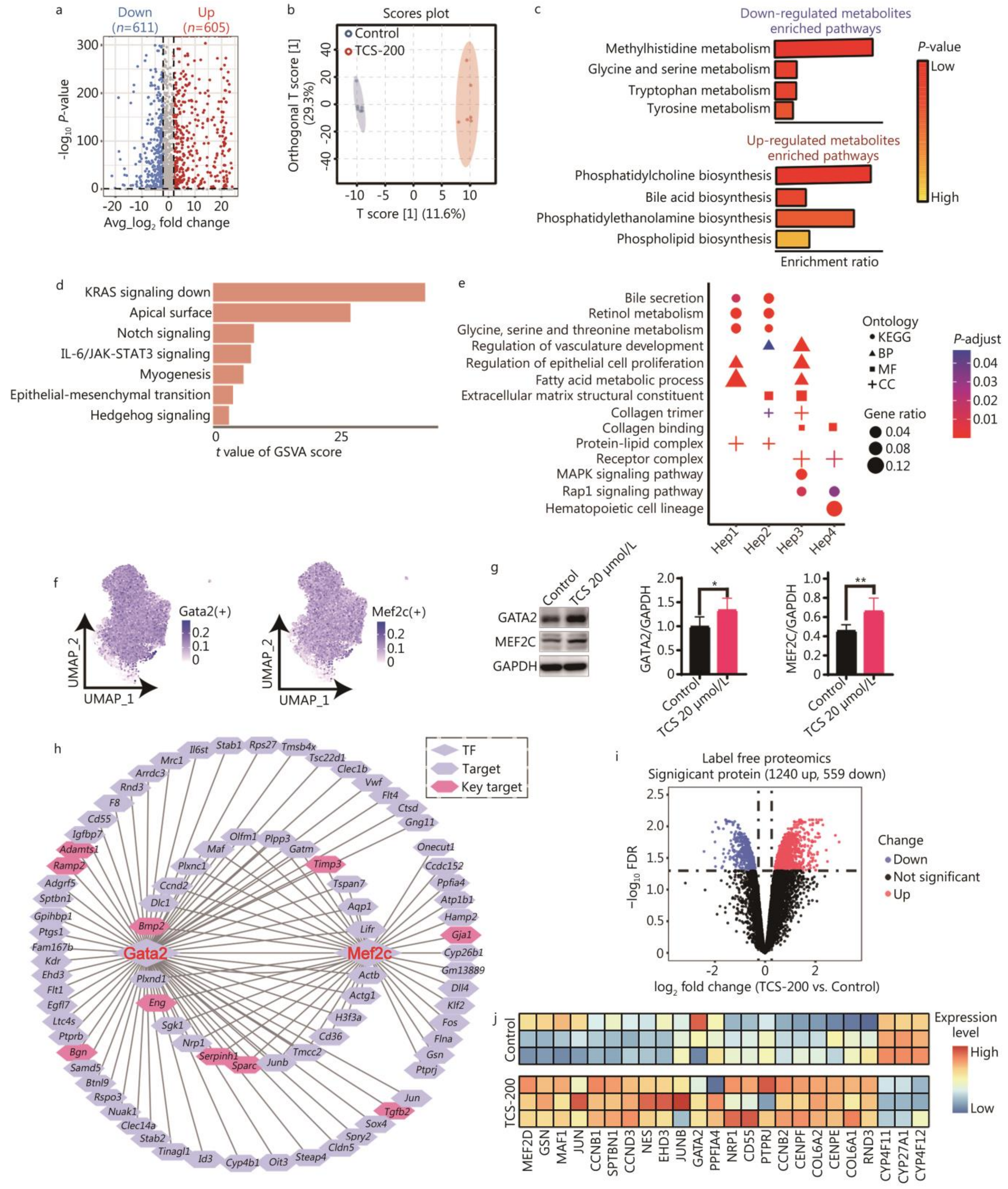

Fig. S5 Cellular change of hepatocytes (Heps). **a** Volcano plot of genes differentially expressed between control and TCS (200 mg/kg)-treated Heps. **b** Ortho PLS-DA analysis of the control and TCS-200 groups in metabolomics data set. **c** Pathway enrichment of down- and up-regulated metabolites. **d** The enriched pathways from GSVA analysis in TCS-200 group. **e** Pathway enrichment of the four subtypes of Heps. The shape represents the type of ontology, color represents adjusted *P*-value, size represents gene ratio. **f** UMAP visualization depicting AUC values

of the expression levels of two selected regulons, i.e., Gata2 and Mef2c. **g** MIHA cells were treated with 20 μmol/L TCS for 24 h and the protein expression levels of GATA2 and MEF2C were detected with Western blotting. GAPDH was used as the loading control. *P < 0.05, **P < 0.01. **h** Transcription regulatory network constructed by Gata2 and Mef2c as well as their target genes. Red text represents TFs, black text for targets (target), pink hexagon for genes related to liver fibrosis (key target). **i** Volcano plot of DEPs between TCS-200 and control groups in proteomics data set. **j** Heatmap showing expression levels of key proteins in TCS-200 and control groups. TCS triclosan, PLS-DA partial least squares discriminant analysis, KEGG Kyoto Encyclopedia of Genes and Genomes, BP biological process, MF molecular function, CC cellular component, GSVA gene set variation analysis, UMAP uniform manifold approximation and projection for dimension reduction, AUC area under the curve, TF transcription factor, DEPs differentially expressed proteins, FDR false discovery rate

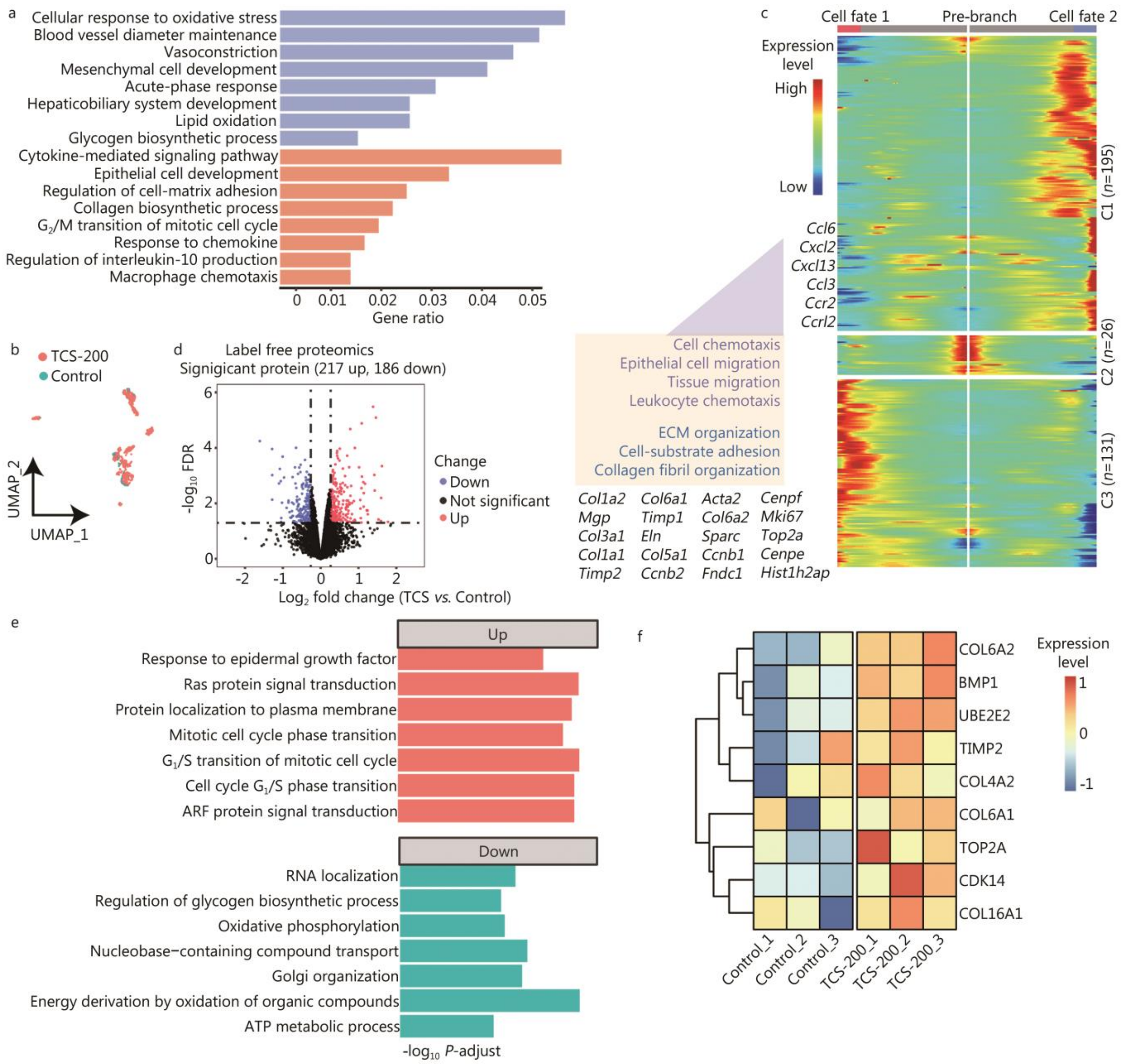

Fig. S6 Cellular change of HSCs. **a** GO biological progresses enriched in down-regulated (top) or up-regulated (bottom) DEGs of HSCs. **b** Distributions distinct subtypes of HSCs in control and TCS-200 groups. **c** BEAM showing genes involved in the development of cell fate 1 and cell fate 2 (C1: cluster 1, C2: cluster 2, C3: cluster 3), and the representative marker genes and enriched GO terms were listed at left. **d** Volcano plot of DEPs between TCS-100, TCS-200 and control groups in proteomics data set. **e** Bar plots showing the biological processes enrich up- and down-regulated DEPs after TCS treatment using proteomics data set. **f** Heatmap showing expression levels of key proteins in TCS-200 and control groups. DEGs differentially expressed genes, HSCs hepatic stellate cells, TCS triclosan, GO Gene Ontology, BEAM branched expression analysis modeling, DEPs differentially expressed proteins, FDR false discovery rate

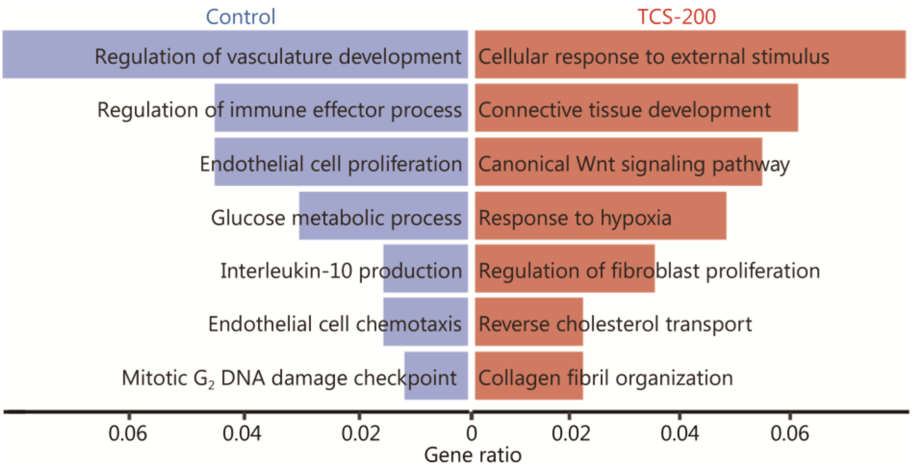

Fig. S7 Functions/processes enriched in DEGs of control (left) and TCS-200 (right) groups in Endos. DEGs differentially expressed genes, TCS triclosan, Endos endothelial cells

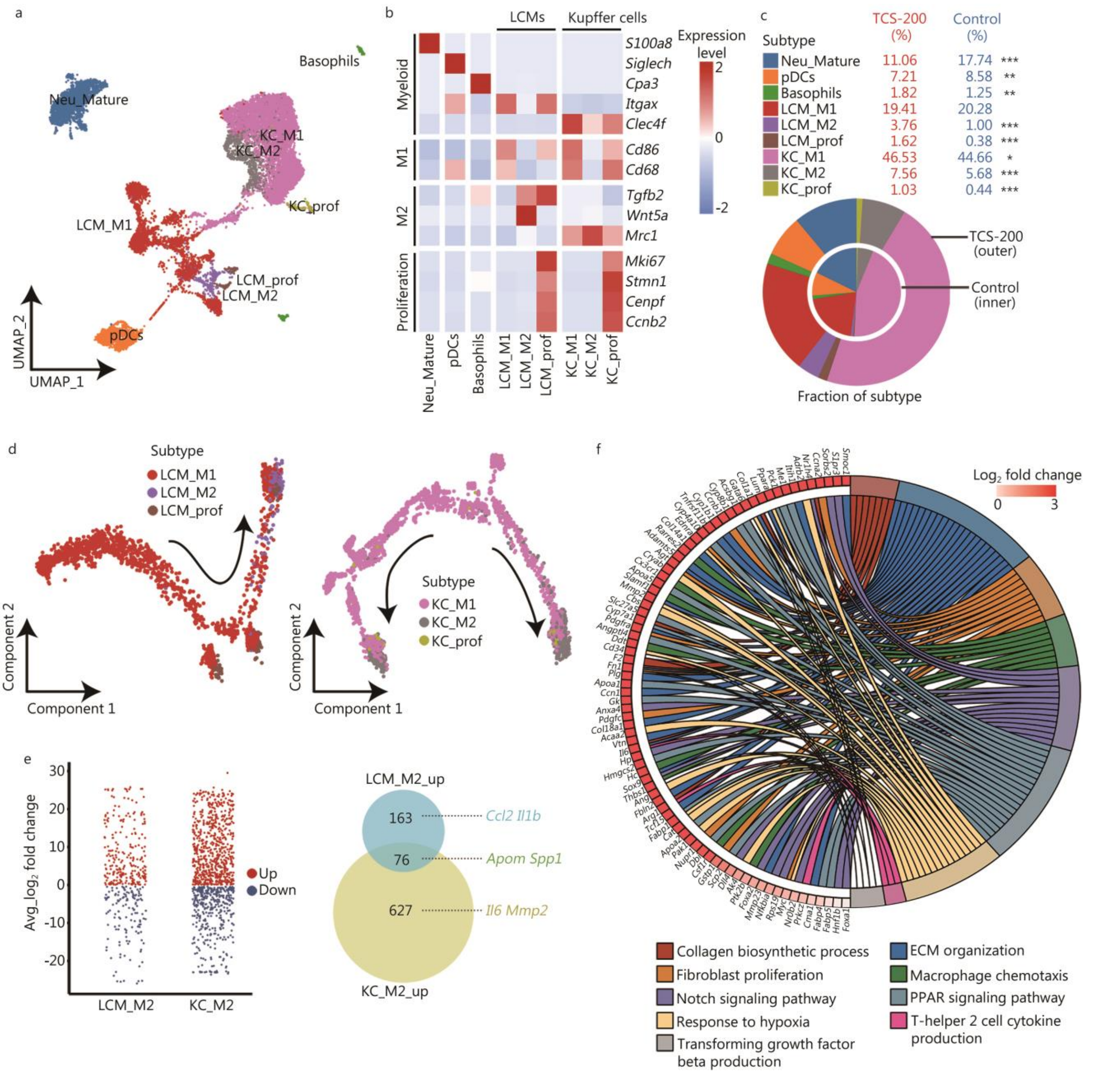

Fig. S8 Emergence of diverse macrophages after TCS treatment. **a** UMAP visualization of distinct subtypes of myeloid cells including Basophils, Neutrophils, pDCs, Kupffer and LCMs. **b** Heatmap of the expression levels of representative markers indicating myeloid, M1, M2 and proliferation functions. **c** Pie chart showing the relative fraction of each myeloid subtype. **d** Pseudotime trajectory indicating the development of LCMs and Kupffer subtypes, respectively, rooting from M1. **e** Strip chart showing the DEGs of LCM_M2 and KC_M2 cells after TCS-200 treatment. Venn diagram displaying the numbers of up-regulated DEGs in the two subtypes, of which the ones associated with liver fibrosis were listed. **f** Chord diagram depicting the pathways enriched in up-regulated genes of KC_M2 subtypes. The left part represents genes ordered by fold change, and the right part represents pathways. Neu_Mature mature neutrophils, pDCs plasmacytoid dendritic cells, LCMs liver capsular macrophages, UMAP uniform manifold approximation and projection for dimension reduction, TCS triclosan, DEGs differentially expressed genes; $^{*}P < 0.05$, $^{**}P < 0.01$, $^{***}P < 0.001$

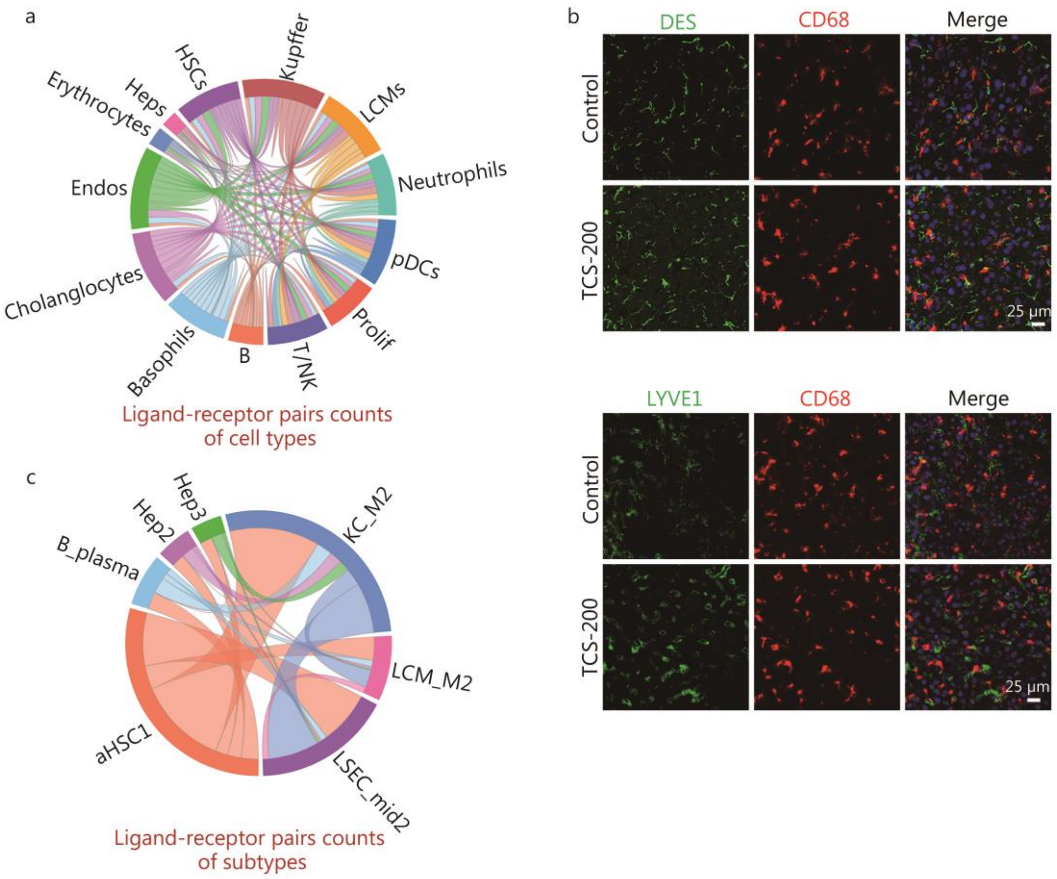

Fig. S9 Cell-cell communication change after TCS treatment. **a** Chord diagram showed the network of cell-cell communication within different cell types. The annotation bar widths are proportional to the sum of pairs counts in each cell type, and edges are proportional to the counts of pairs between cell types. **b** Co-location of (top) HSCs (DES: green) and macrophage (CD68: red), and (bottom) liver sinusoidal endothelial cells (LYVE1: green) and macrophage (CD68: red) by immunofluorescence staining. Scale bar = 25 μm. **c** Chord diagram showed the network of cell-cell communication within the selected crucial subtypes. Endos endothelial cells, Heps hepatocytes, HSCs hepatic stellate cells, aHSC1 activated hepatic stellate cell subtype 1, LCMs liver capsular macrophages, pDCs plasmacytoid dendritic cells, Prolif proliferative cells, T/NK T/natural killer cells, TCS triclosan, DES desmin, LYVE1 lymphatic vessel endothelial hyaluronan receptor 1
